# Phylogenomics, divergence time estimation, and biogeography of *Iris* species from Kazakhstan using plastome sequence analysis

**DOI:** 10.3389/fpls.2026.1860819

**Published:** 2026-06-17

**Authors:** Shyryn Almerekova, Moldir Yermagambetova, Aruzhan Alikhanova, Daniyar Yerbolatov, Bektemir Osmonali, Yerlan Turuspekov

**Affiliations:** 1 Molecular Genetics Laboratory, Institute of Plant Biology and Biotechnology, Almaty, Kazakhstan; 2 Faculty of Biology and Biotechnology, Al Farabi Kazakh National University, Almaty, Kazakhstan; 3 Plant World Cadastre Laboratory, Institute of Botany and Phytointroduction, Almaty, Kazakhstan

**Keywords:** biogeography, Iris, microsatellites, molecular dating, phylogeny, plastome

## Abstract

**Introduction:**

The genus *Iris* Tourn. ex L. (Iridaceae Juss.) comprises more than 300 accepted species distributed across the Northern Hemisphere and widely valued for its medicinal and ornamental significance. However, *Iris* is a taxonomically complex genus with controversial circumscription and unresolved phylogenetic relationships, emphasizing the need for comprehensive systematic studies.

**Methods:**

In this study, plastomes of 14 *Iris* species collected in Kazakhstan were sequenced, assembled, and annotated. Comparative genomic analyses, divergence time estimation, and biogeographic reconstruction were performed based on complete plastome data.

**Results:**

All plastomes exhibited the typical quadripartite circular structure, with genome sizes ranging from 150, 612 bp (*I. tenuifolia*) to 155, 046 bp (*I. kuschakewiczii*). The gene content was highly conserved among the studied species, with 133 genes identified, including 87 protein-coding genes (PCGs), 38 tRNA genes, and eight rRNA genes. Among these, eight PCGs (*ndhB, rpl2, rpl23, rps7, rps12, rps19, ycf1*, and *ycf2*), seven tRNA genes (*trnA-UGC, trnH-GUG, trnIGAU, trnL-CAA, trnN-GUU, trnR-ACG*, and *trnV-GAC*), and all four rRNA genes (*rrn4.5, rrn5, rrn16*, and *rrn23*) were duplicated. Ten highly variable regions were identified, namely *rps16, rps16-trnQ(UUG), trnS(GCU)-trnG(UCC), trnG(UCC), trnY(GUA), trnD(GUC), rpl32, rps15, clpP*, and *ycf1*, most of which were located in the large single-copy region. A total of 2, 401 simple sequence repeats (SSRs) were detected across the 14 plastomes, with an average of 171.5 SSRs per species. Mononucleotide repeats were the most abundant type, accounting for 65.7% of all SSRs, with a strong bias toward A/T motifs. Phylogenetic analyses resolved three major clades corresponding to the subgenera *Iris*, *Scorpiris, Limniris*, and *Hermodactyloides*. Molecular dating suggested that *Iris* originated approximately 49.31 Ma (95% CI: 40.13–59.60 Ma), with crown group diversification beginning around 41.43 Ma (95% CI: 33.97–50.97 Ma). Ancestral area reconstruction indicated a combined Eastern Asia-Central Asia origin for the genus.

**Discussion:**

Overall, this study provides comprehensive plastome-based insights that may contribute to resolving phylogenetic relationships, improving population genetic studies, and elucidating the evolutionary history and biogeography of *Iris*.

## 1 Introduction

The genus *Iris* Tourn. ex. L. belongs to tribe Irideae Kitt., subfamily Iridoideae Eaton, family Iridaceae Juss. with 312 accepted species (POWO, 2026). It is the largest genus within the family and is widely distributed throughout the Northern Hemisphere (Mathew, 1989; Choi and Lee, 2024). In Kazakhstan, the genus is represented by approximately 30 species, nine of which are listed in the national Red Data Book (Baitulin, 2014). *Iris* species are of considerable horticultural importance as decorative plants, mainly due to their striking floral diversity and wide range of flower colors. These traits are associated with the accumulation of pigments such as anthocyanins and carotenoids (Roguz et al., 2020; Crișan, 2025). The rhizomes of *Iris* species possess medicinal properties, including antioxidant, anticarcinogenic, anti-inflammatory, and antimicrobial activities (Benoit-Vical et al., 2003; Wollenweber et al., 2003; Shin et al., 2011; Nazir, 2013; Bensari, 2020; Mykhailenko et al., 2020). The genus is also important because of its applications in culinary, perfumery, and cosmetics (Cai et al., 2021; Öztaş et al., 2024; Crișan, 2025). Moreover, several *Iris* species have a profound symbolic significance and serve as national emblems or cultural icons for various countries worldwide (Radanova, 2023).

Despite its biological and economic importance, *Iris* remains a taxonomically complex genus with a controversial circumscription and unresolved phylogenetic relationships, highlighting the need for comprehensive systematic studies (Jiang et al., 2018; Kang et al., 2020; Feng et al., 2022; Sennikov et al., 2023; Choi and Lee, 2024). Traditional classifications based primarily on morphological traits have undergone multiple revisions, particularly regarding subgeneric and sectional delimitations within Iridaceae (Wilson, 2011; Mavrodiev et al., 2014; Wilson et al., 2016; Jiang et al., 2018; Boltenkov and Artyukova, 2024). The most widely accepted system, proposed by Mathew (Mathew, 1989), recognizes six subgenera (*Iris, Limniris* (Tausch) Spach*, Nepalensis* (Dykes) Lawr., *Xiphium* (Mill.) Spach, *Scorpiris* Spach, and *Hermodactyloides* Spach), along with 12 sections and 16 series within subgenus *Limniris*. This classification builds upon earlier works by Dykes (Dykes, 1913), Lawrence (Lawrence, 1953), and Rodionenko (Rodionenko, 1987). Subgeneric classification relies on several morphological traits: the type of the geophytic organ, aril development on seeds, the formation of sepal crests, and the presence of well-defined sepal hairs (beards) (Wilson, 2011). Molecular studies have significantly advanced the understanding of molecular identification and phylogeny within the genus *Iris* (Wilson, 2011; Wilson, 2017). Furthermore, with the development of modern molecular methods, findings that do not support the conventional classification have been obtained (Guo and Wilson, 2013; Mavrodiev et al., 2014; Crespo et al., 2015; Tojibaev et al., 2025).

Over the past two decades, taxonomic and phylogenetic studies of *Iris* have largely relied on short regions of the plastid genome (Vereecken et al., 2012; Guo and Wilson, 2013; Wilson, 2017; Jiang et al., 2018; Boltenkov and Artyukova, 2024; Chen et al., 2024; Apostol et al., 2024). With the advent of next-generation sequencing technologies, complete plastid genomes have emerged as powerful tools for resolving complex phylogenetic relationships because of their conserved structure, uniparental inheritance, and low recombination rates (Huang et al., 2022a, Huang et al. 2022b; Li et al., 2023; Xu et al., 2023). The chloroplast is a double-membrane organelle essential for higher plants, containing its own genome with a conserved quadripartite structure comprising a large single-copy (LSC) region, a small single-copy (SSC) region, and two inverted repeat (IR) regions (Daniell et al., 2016; Duan et al., 2022). Plastome sequencing has become widely used in plant systematics and phylogenetics due to its relatively compact size and informative genomic content (Zhang et al., 2023; Kassem, 2025; Song et al., 2024a). Recent plastome-based studies (Kang et al., 2020; Feng et al., 2022; Choi and Lee, 2024) have significantly improved our understanding of evolutionary relationships within *Iris*, revealing conserved genome organization and identifying highly variable regions useful for phylogenetic inference. Moreover, whole plastome analyses have enabled more robust reconstruction of evolutionary history and clarified relationships among subgenera and sections, including complex groups such as *Scorpiris* (Ortikov et al., 2023) and *Oncocyclus* (Volis et al., 2024). Nevertheless, despite the increasing availability of plastid genome data, phylogenetic relationships within *Iris* remain incompletely resolved, and further comparative genomic studies are needed to identify informative molecular markers and clarify evolutionary relationships within the genus.

Population genetic studies in the genus *Iris* remain comparatively underdeveloped, primarily due to the limited availability of robust and transferable molecular markers. To date, genetic diversity in *Iris* has been assessed using a range of marker systems, including amplified fragment length polymorphisms (AFLP) (Volis et al., 2022), single-nucleotide polymorphisms (SNPs) (Hamlin and Arnold, 2014; Cohen and Turgman-Cohen, 2023), inter-simple sequence repeats (ISSR) (Rasul et al., 2025; Bo et al., 2025), start codon-targeted (SCoT) (Verma et al., 2022; Majeed et al., 2024), sequence-related amplified polymorphism (SRAP) (Majeed et al., 2024; Bo et al., 2025), and simple sequence repeats (SSRs) (Sun et al., 2012; Weber et al., 2020; Zhang et al., 2021). Among these, SSRs (microsatellites) are regarded as highly informative co-dominant markers due to their high allelic diversity, reproducibility, and genome-wide distribution, making them particularly suitable for fine-scale population genetic analyses (Terzoli et al., 2014; Xu et al., 2023). SSR markers provide powerful tools for quantifying genetic diversity, population structure, and gene flow, thereby offering critical insights into evolutionary dynamics (Peng et al., 2024; Hai et al., 2025). Such information is essential for the development of evidence-based conservation strategies and the effective management of plant genetic resources. However, despite their advantages, the development and application of SSR markers in *Iris* remain limited, constraining comprehensive population-level studies. Therefore, the identification and characterization of novel SSR loci are necessary to facilitate future research on genetic diversity, conservation genetics, and the breeding of economically and ecologically important *Iris* species.

In Central Asia, *Iris* species have been studied primarily from biogeographical (Shomurodov et al., 2021; Sennikov et al., 2023; Alikhanova et al., 2025; Tojibaev et al., 2025), phytochemical (Omarova et al., 2024; Aitkenova et al., 2025; Ramazanova et al., 2026), and morphological (Turgunov et al., 2019; Rakhimova et al., 2020; Ramazanova et al., 2020) perspectives. Recent phylogeny-based classification of *Iris* s.l. in the Mountains of Central Asia biodiversity hotspot (Sennikov et al., 2023) has significantly improved understanding of species diversity in the region by providing an updated checklist and incorporating modern phylogenetic and nomenclatural data. However, despite these advances, the genus still requires further taxonomic revision to fully resolve its complex classification.

In this study, we analyzed the complete plastome sequences of 14 *Iris* species with the aim of (i) performing a comparative analysis of plastomes; (ii) identifying hotspot regions as potential molecular markers; (iii) reconstructing phylogenetic relationships using different methods; and (iv) estimating biogeography and divergence times. The obtained genomic data will contribute to a better understanding of phylogenetic relationships within *Iris* and support the development of a more robust classification framework for the family Iridaceae.

## 2 Materials and methods

### 2.1 Sample collection and DNA extraction

**Figure 1:**
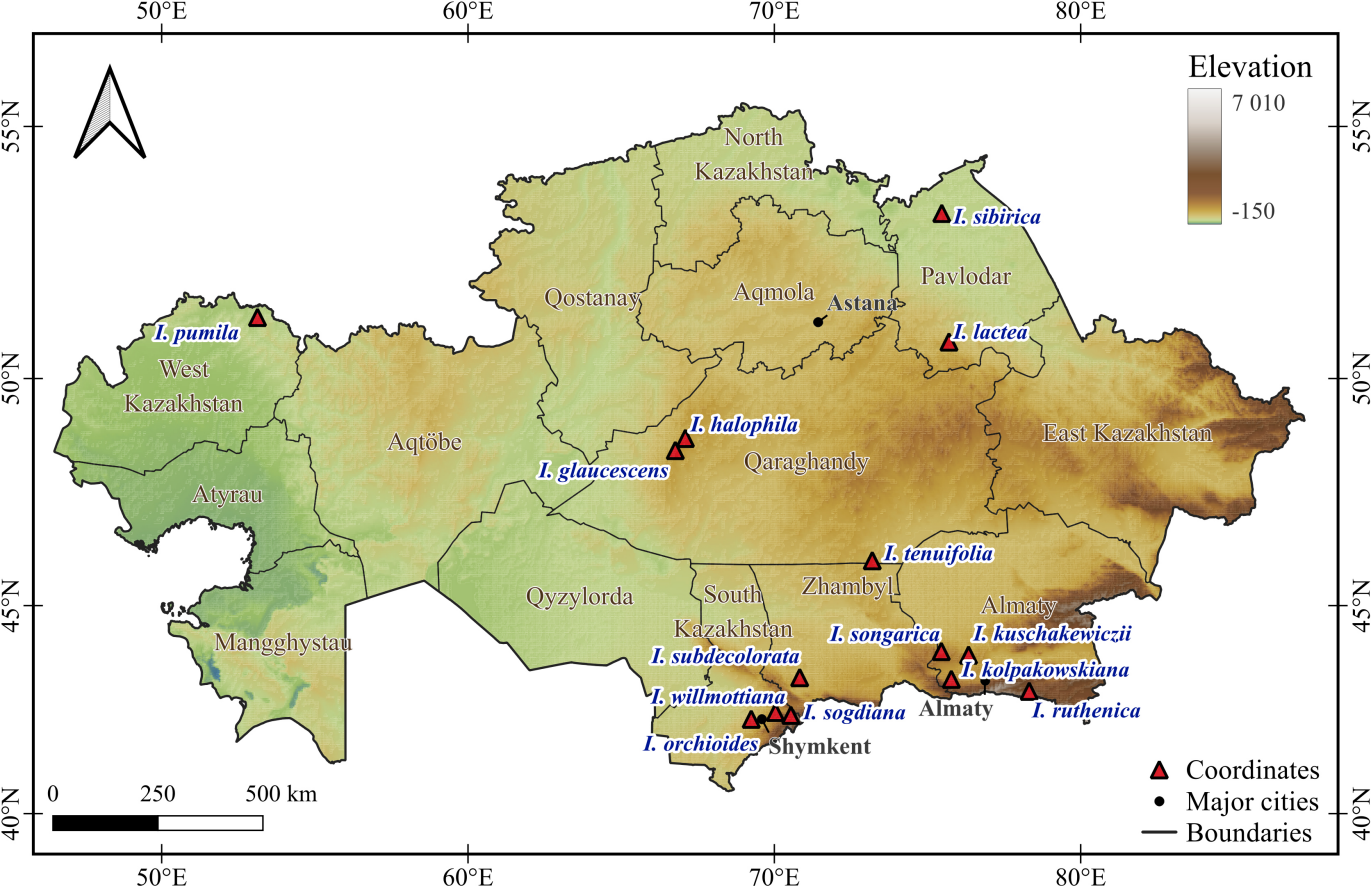
Geographic distribution and sampling locations of *Iris* species included in this study across Kazakhstan. Sampling sites are indicated by red triangles. Major cities are shown as black dots, and administrative boundaries are outlined. Elevation is represented by a color gradient (meters above sea level). Species names are labeled at their corresponding collection localities.

Samples of all 14 *Iris* species (*I. glaucescens* Bunge, *I. halophila* Pall., *I. lactea* Pall., *I. pumila* L., *I. ruthenica* Ker Gawl., *I. sibirica* L., *I. sogdiana* Bunge, *I. songarica* Schrenk ex Fisch. & C.A.Mey., *I. tenuifolia* Pall., *I. kolpakowskiana* Regel, *I. kuschakewiczii* B.Fedtsch., *I. orchioides* Carrière, *I. subdecolorata* Vved., *I. willmottiana* Foster) were collected and identified by the authors from field observations conducted in Kazakhstan during 2017, 2024, and 2025. The species were identified based on their key diagnostic features, following the fundamental floristic works, such as Flora of Kazakhstan (Flora of Kazakhstan, 1958), Flora of the USSR (Flora of the USSR, 1935), and Flora of Siberia (Malyshev and Peshkova, 1987). The detailed morphological characteristics of 14 *Iris* species were provided in **Supplementary Table 1**. The plant leaves were collected from different regions of Kazakhstan. Detailed information on collection sites was given in **Figure 1** and **Supplementary Table 2**. Permission to collect plant leaves was obtained from the Forestry and Wildlife Committee of the Ministry of Ecology, Geology, and Natural Resources of the Republic of Kazakhstan. The voucher specimens were deposited in the herbarium of the Institute of Plant Biology and Biotechnology (IPBB) (**Supplementary Table 2**). Fresh leaves were obtained from the field, dried, and stored in silica gel. Total genomic DNAs were extracted according to the cetyltrimethylammonium bromide (CTAB) method (Doyle and Doyle, 1987). Then, the extracted DNA was examined on a 1% agarose gel and using the NanoDrop 2000 instrument (Thermo Fisher Scientific Inc., USA). For each of the 14 species, one representative accession was selected for plastome sequencing and downstream comparative genomic analyses.

### 2.2 Plastome sequencing, assembly, and annotation

The DNA libraries for plastome sequencing were constructed using the TruSeq Nano DNA kit (Illumina Inc., San Diego, CA, USA). High-throughput sequencing of 14 *Iris* species plastomes was carried out by generating two sets of 150 bp paired-end raw reads per sample on the Illumina NovaSeq X platform (Illumina Inc., San Diego, CA, USA) at Macrogen Inc. (Seoul, Republic of Korea). The generated raw data were QC filtered and trimmed using the Trimmomatic v0.39 software (Bolger et al., 2014). Sequencing quality was high across all samples, with Q30 values ranging from 94.8% to 98.3% after filtering. Subsequent assembly of the filtered reads was performed using SPAdes 3.15.5 (Bankevich et al., 2012). Plastome coverage ranged from 99.97% to 100%, while average mapping depth ranged from 295× to 1911×. The complete plastome was annotated using GeSeq v2.03 (Tillich et al., 2017), followed by manual verification against published plastomes of *Iris* species. A detailed circular map of the plastome for 14 *Iris* species was generated using OGDRAW v1.3.1 (Greiner et al., 2019). The sequences obtained in this study were deposited in the NCBI GenBank under accession numbers PX505513-PX505515, PX505517-PX505522, PX505524-PX505525, PX915248-PX915249, and PX910022. The raw data are available under BioProject PRJNA1471469 (**Supplementary Table 2**).

### 2.3 Plastome comparison, boundary regions comparison, and simple sequence repeats identification

The plastomes of 14 *Iris* species were aligned with the mVISTA program (Frazer et al., 2004) in Shuffle-LAGAN mode. The extents of IRs, SSCs, and LSCs in the plastomes were compared using IRscope software (https://irscope.shinyapps.io/irapp). The DnaSP v6.12.03 program (Librado and Rozas, 2009) was employed to identify hypervariable regions and nucleotide diversity of plastomes, with a 200 bp step size and a 600 bp window length.

Simple sequence repeats (SSRs) were identified using MIcroSAtellite (MISA) version 2.1 (https://webblast.ipk-gatersleben.de/misa) with the following thresholds: eight for mononucleotide repeats, four for dinucleotide and trinucleotide repeats, and three for tetranucleotide, pentanucleotide, and hexanucleotide repeats.

### 2.4 Codon usage

To investigate synonymous codon usage patterns across the 14 *Iris* plastomes, the relative synonymous codon usage (RSCU) was calculated for protein-coding genes (PCGs) extracted using CodonW v1.4.2 (http://codonw.sourceforge.net/). As short sequences could lead to inaccurate codon interpretations, PCGs with a length of at least 300 bp were selected for further analysis. RSCU values indicate the ratio of the observed frequency of a codon to the theoretically expected frequency if all synonymous codons for the same amino acid were used without preference. The value of RSCU more than 1.0 means that it is a high-frequency preferred codon, whereas a value less than 1.0 indicates a low-frequency unpreferred codon, and a value equal to 1.0 means there is no usage bias. The resulting RSCU plot was visualized as a stacked bar chart, grouped by amino acid, using R software v4.5.1 (R Core Team, 2020).

### 2.5 Phylogenetic analysis

The 14 plastomes assembled in this study, together with 18 publicly available *Iris* plastomes retrieved from the National Center for Biotechnology Information (NCBI) GenBank database, were used to infer phylogenetic relationships within the genus. In total, 32 ingroup taxa and two outgroup species (*Moraea polystachya* and *M. spathulata*) were included in the phylogenetic analyses, which were conducted using both maximum likelihood (ML) and Bayesian inference (BI) approaches. Multiple sequence alignment was performed using MAFFT v7.525 with default parameters. The ML phylogenetic tree was reconstructed in IQ-TREE v2.2.2.6 (Minh et al., 2020) under the best-fit nucleotide substitution model TVM+F+I+R3 with branch support assessed using 1, 000 bootstrap replicates. Bayesian inference was carried out using MrBayes v3.2.6 (Ronquist et al., 2012), with two independent Markov chain Monte Carlo (MCMC) runs of 25, 000, 000 generations each. Trees were sampled every 100 generations, and the first 25% of sampled trees were discarded as burn-in. The Neighbor-Joining (NJ) tree was generated from aligned fasta file in MEGA v12.1 (Kumar et al., 2024) software with 1, 000 bootstrap replicates. The resulting phylogenetic trees were visualized and edited using FigTree v1.4.4 (Rambaut, 2014). Subgeneric classification follows the system proposed by Mathew (1989), as supported and further refined by subsequent studies (Crespo et al., 2015; Nikitina et al., 2023; Chen et al., 2024; Parnikoza et al., 2017; Volis et al., 2024; Alexeeva, 2018; Boltenkov and Artyukova, 2024).

### 2.6 Divergence time estimation and ancestral area reconstruction

Divergence time was estimated using MCMCtree in PAML v4.9 (Yang, 2007). Before the analysis, we observed substantial number of loci with low phylogenetic signal, thus to reduce computational burden we excluded coding sequences (CDSs) with fewer than 10% parsimony-informative sites, resulting in a final dataset of 65 CDSs from 88 *Iris* species. These loci were concatenated using AMAS v1.0 (Borowiec, 2016). A guide tree topology was inferred in IQ-TREE 3 using the MFP+MERGE, -alrt 1000, and -B 1000 options. The mean substitution rate was estimated in BASEML as 0.062449 per 10^8 years. Based on this estimate, the prior for the overall substitution rate (rgene_gamma) was set to G(2, 32), and the prior for the rate-drift parameter (sigma2_gamma) was set to G(1, 10). An independent-rates relaxed clock model (clock = 2) was applied. The birth-death parameters were set to 1, 1, 0, m. The MCMC analysis was run for a total of 11, 000, 000 generations, with a burn-in of 1, 000, 000 generations, samples taken every 100 generations (resulting in 100, 000 samples for posterior inference). Convergence was assessed using Tracer v1.7.2 (Rambaut et al., 2018), and all parameters had effective sample sizes (ESS) greater than 200. As genus *Iris* and entirely Iridaceae family do not possess any fossil evidences. We used more possible calibrations as suggested by Sauquet (2013) and Duchêne et al. (2014). The MCMCtree needs the root to be calibrated, thus root was constrained using a secondary calibration point for the stem age of Orchidaceae (102–120 Ma) (Givnish et al., 2016). The crown age of *Astelia* was constrained to 23.2 Ma (Maciunas et al., 2011), the crown age of Hemerocallidoideae to 38.0-47.8 Ma (Conran et al., 2003), and the stem age of Iridaceae to 73.0-94.5 Ma (Joyce et al., 2018).

Occurrence data for *Iris* species were obtained from the Plants of the World Online (POWO, 2026). Species distributions were coded into the following biogeographic regions based on their current ranges: Eastern Asia (A), South & Southeast Asia (B), Central Asia (C), the Irano-Turanian region (D), Europe & Mediterranean (E), Levant (F), and North America (G). Ancestral area reconstruction was performed in RASP v4.1 (Yu et al., 2020). The tree file generated by MCMCtree was used as input to compare alternative BioGeoBEARS models. The best-fitting model was selected based on the highest Akaike information criterion (AIC) weight.

## 3 Results

### 3.1 Plastome features

**Figure 2:**
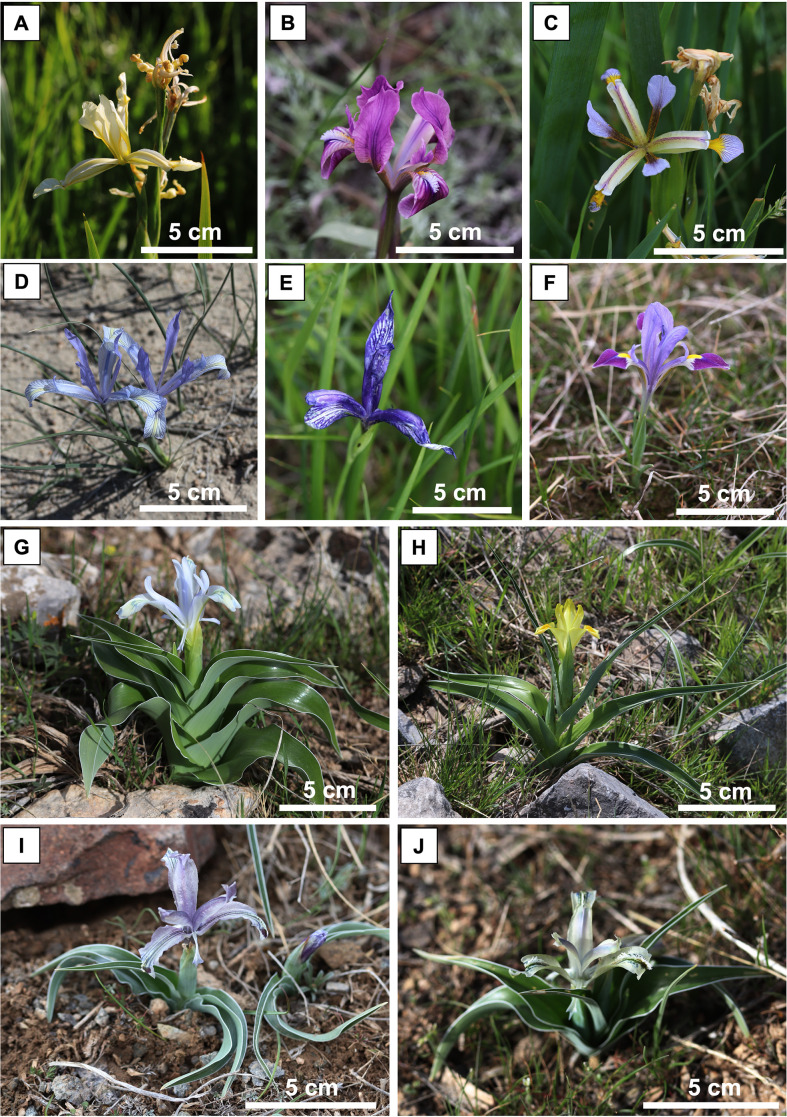
Representative photographs of *Iris* species in their natural habitats: **(A)** *Iris halophila*; **(B)** *Iris pumila*; **(C)** *Iris sogdiana*; **(D)** *Iris tenuifolia*; **(E)** *Iris ruthenica*; **(F)** *Iris kolpakowskiana*; **(G)** *Iris willmottiana*; **(H)** *Iris orchioides*; **(I)** *Iris kuschakewiczii*; and **(J)** *Iris subdecolorata.*.

The plastomes of the 14 studied *Iris* species (**Figure 2**) exhibited a typical quadripartite circular structure, consisting of a large single-copy (LSC), a small single-copy (SSC), and a pair of inverted repeat (IR) regions. The total plastome size ranged from 150, 612 bp to 155, 046 bp, including the LSC region (80, 726–82, 656 bp), SSC region (17, 569–18, 464 bp), and IR regions (50, 896–54, 534 bp). The overall GC content was relatively consistent across the analyzed *Iris* plastomes, ranging from 37.8% to 38.9%. A total of 133 genes were identified, comprising 87 PCGs, 38 tRNA genes, and 8 rRNA genes (**Table 1**).

**Table 1:** Information on plastome features of 14 *Iris* species.

Species	Genome length (bp)	Genes (duplicated)	Protein genes (duplicated)	tRNA genes (duplicated)	rRNA genes (duplicated)	GC content (%)	LSC region length (bp)	SSC region length (bp)	IR region length (bp)	GenBank accession number
*I. glaucescens*	153, 453	133 (19)	87 (8)	38 (7)	8 (4)	37.9	82, 599	18, 438	52416	PX505513
*I. halophila*	152, 390	133 (19)	87 (8)	38 (7)	8 (4)	38.1	81, 883	18, 153	52354	PX505514
*I. lactea*	152, 517	133 (19)	87 (8)	38 (7)	8 (4)	38.9	82, 376	18, 097	52044	PX505515
*I. pumila*	153, 550	133 (19)	87 (8)	38 (7)	8 (4)	37.8	82, 656	18, 464	52430	PX505517
*I. sibirica*	152, 396	133 (19)	87 (8)	38 (7)	8 (4)	38.1	81, 880	18, 262	52254	PX505518
*I. sogdiana*	152, 410	133 (19)	87 (8)	38 (7)	8 (4)	38.1	81, 909	18, 147	52354	PX505519
*I. songarica*	153, 002	133 (19)	87 (8)	38 (7)	8 (4)	38.1	81, 329	17, 798	53875	PX505520
*I. tenuifolia*	150, 612	133 (19)	87 (8)	38 (7)	8 (4)	38.1	80, 726	18, 072	51814	PX505521
*I. ruthenica*	152, 298	133 (19)	87 (8)	38 (7)	8 (4)	38.2	82, 388	18, 152	51758	PX915248
*I. willmottiana*	151, 278	133 (19)	87 (8)	38 (7)	8 (4)	38.0	82, 196	18, 168	50914	PX505522
*I. orchioides*	151, 621	133 (19)	87 (8)	38 (7)	8 (4)	38.0	82, 240	18, 325	51056	PX505524
*I. kuschakewiczii*	155, 046	133 (19)	87 (8)	38 (7)	8 (4)	38.1	82, 274	18, 238	54534	PX505525
*I. subdecolorata*	151, 365	133 (19)	87 (8)	38 (7)	8 (4)	38.0	82, 231	18, 238	50896	PX915249
*I. kolpakowskiana*	151, 387	133 (19)	87 (8)	38 (7)	8 (4)	38.0	81, 150	17, 569	52668	PX910022

**Figure 3:**
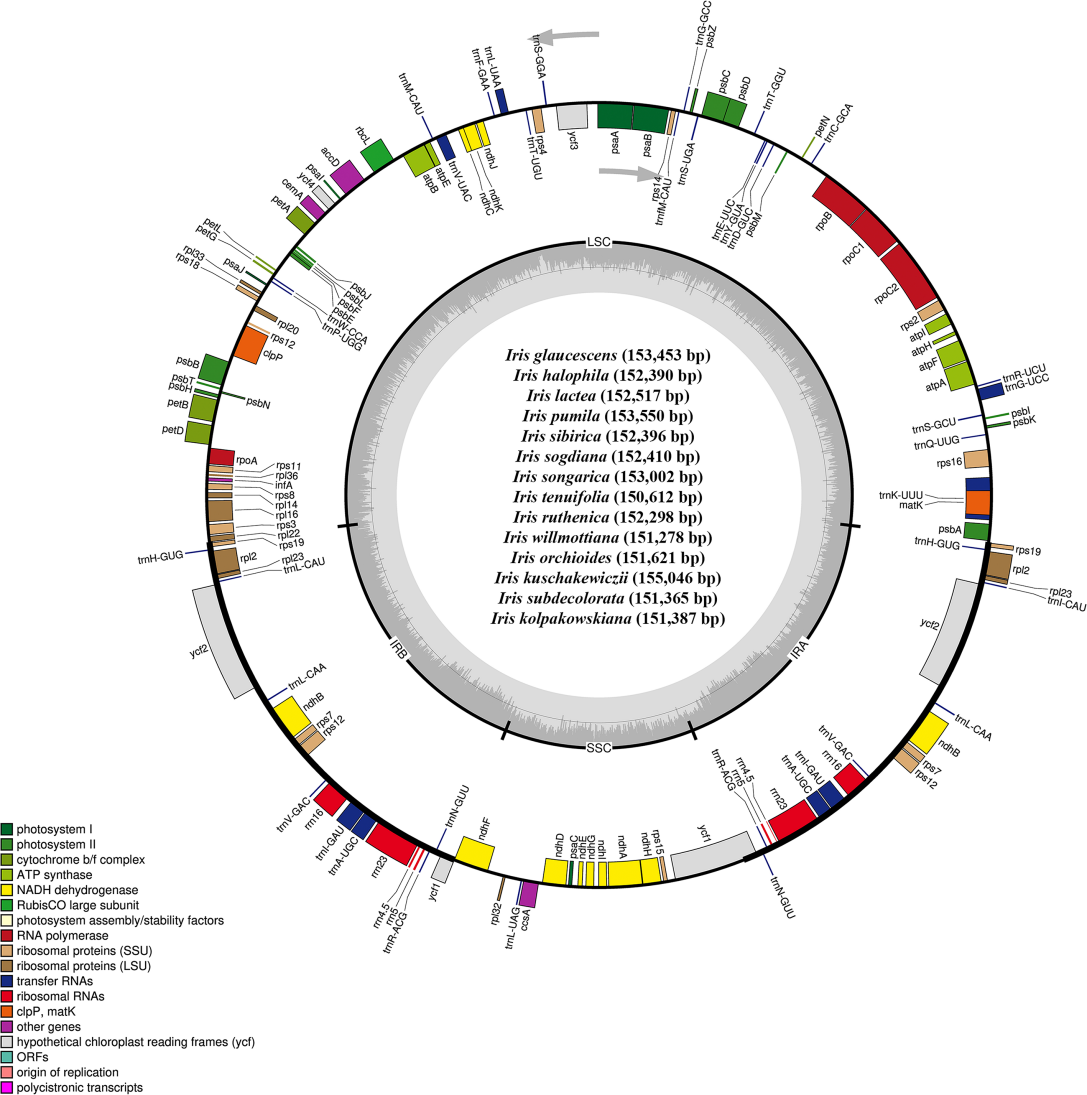
Plastome map of the 14 *Iris* species analyzed in this study. Genes located on the inner circle are transcribed in the clockwise direction, whereas those on the outer circle are transcribed counterclockwise. Genes are color-coded based on their functional categories. In the inner circle, darker gray bars represent GC content, while lighter gray bars indicate AT content. IR, inverted repeat; SSC, small single-copy region; LSC, large single-copy region.

The eight PCGs (*ndhB, rpl2, rpl23, rps7, rps12, rps19, ycf1*, and *ycf2*), seven tRNA genes (*trnA-UGC, trnH-GUG, trnI-GAU, trnL-CAA, trnN-GUU, trnR-ACG*, and *trnV-GAC*), and all four rRNA genes were present in duplicate. Among the annotated genes, 15 (*trnA-UGC, trnG-UCC, trnI-GAU, trnK-UUU, trnL-UAA, trnV-UAC, rps16, rpl16, rpl2, rpoC1, atpF, petB, petD, ndhA*, and *ndhB*) contained a single intron, whereas three genes (*rps12, clpP*, and *ycf3*) possessed two introns (**Figure 3**; **Supplementary Table 3**).

### 3.2 IR expansion and contraction

**Figure 4:**
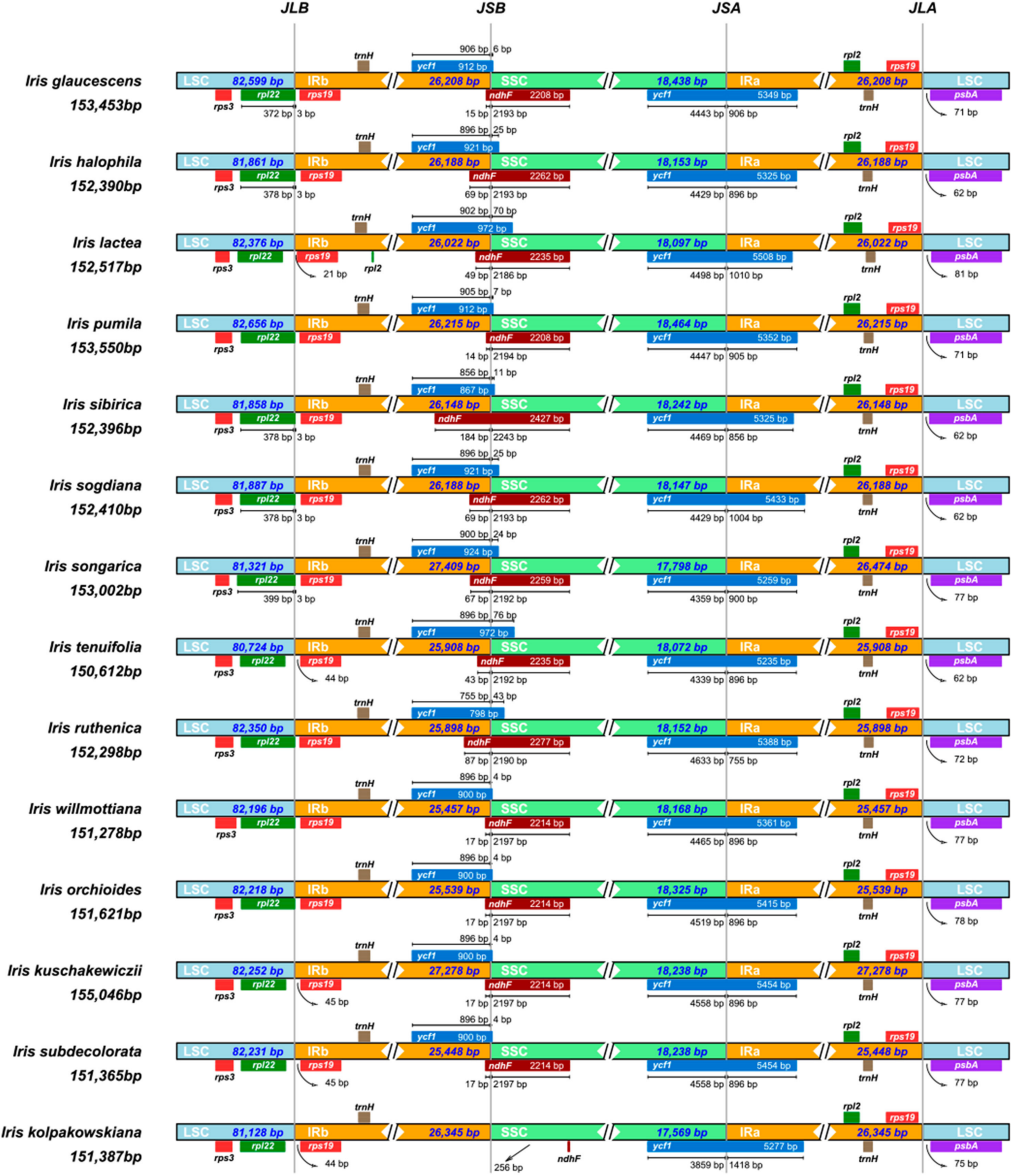
Comparative analysis of the junctions between the large single-copy (LSC), small single-copy (SSC), and inverted repeat regions (IRa and IRb) in 14 *Iris* plastomes. Junctions are denoted as JLB (LSC/IRb), JSB (IRb/SSC), JSA (SSC/IRa), and JLA (IRa/LSC).

The structure and boundary regions of the plastomes from 14 *Iris* species were compared, with particular emphasis on the junctions between the LSC, SSC, and IR regions. The total plastome length ranged from 150, 612 bp (*I. tenuifolia*) to 155, 046 bp (*I. kuschakewiczii*), indicating a high degree of genome-size conservation across the genus. The positions of the four junctions (JLB, JSB, JSA, and JLA) were largely conserved among the examined species, with only minor variation in the extent of adjacent genes. The *ycf1* gene consistently spanned the SSC/IR boundaries, with a portion duplicated within the IR regions, resulting in a truncated copy at the IRb/SSC junction. Similarly, the *ndhF* gene was located near the SSC/IRb boundary, exhibiting slight differences in overlap or distance from the junction among species. At the LSC/IRb junction, the *rps19* gene was positioned close to or slightly extended into the IRb region, whereas *rpl22* and *rps3* were entirely located within the LSC region. At the IRa/LSC boundary, the *trnH* gene was consistently located in the LSC region near the junction, while the *psbA* gene was situated further downstream (**Figure 4**).

### 3.3 Plastome comparison and hotspot identification analysis

**Figure 5:**
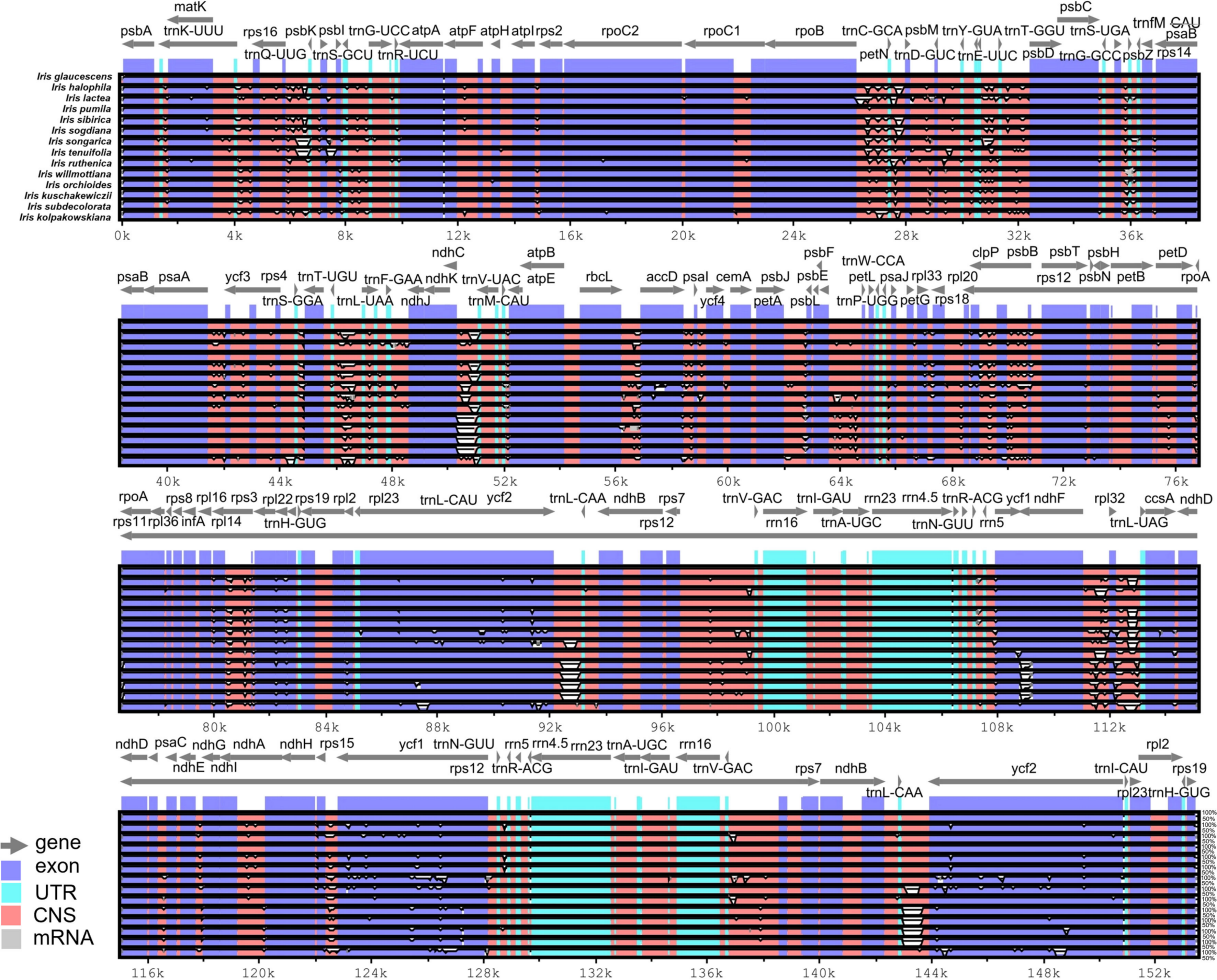
Comparative alignment of 14 *Iris* plastomes visualized using mVISTA. The x-axis shows nucleotide positions along the plastome, while the y-axis represents sequence identity percentages (50–100%). Gray arrows indicate gene locations and directions.

A comparative analysis of *Iris* plastomes was performed using mVISTA and subsequently visualized. The results indicated that the LSC region exhibited higher variability than the SSC and IR regions. In general, non-coding regions showed greater sequence divergence than coding regions across the analyzed plastomes (**Figure 5**).

**Figure 6:**
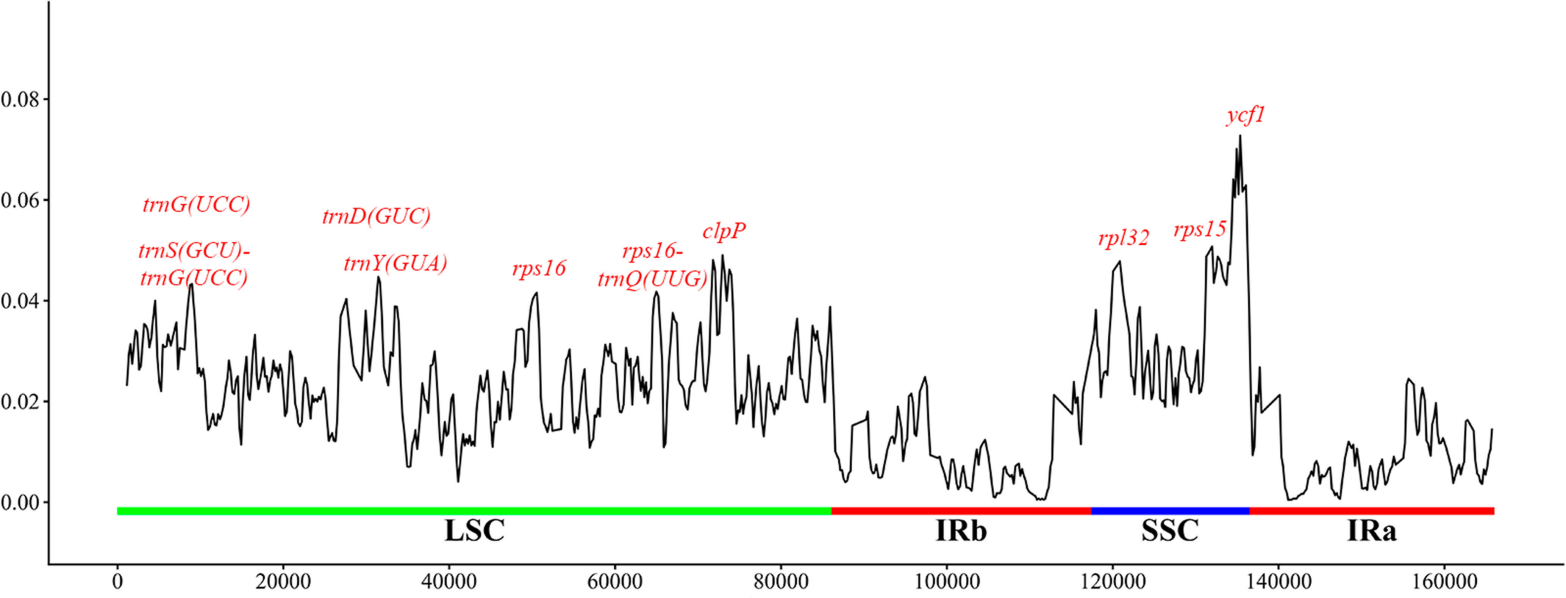
Sliding window analysis of the complete plastomes of 14 *Iris* species (window length: 600 bp; step size: 200 bp). The x-axis represents the midpoint position of each window, and the y-axis indicates nucleotide diversity (Pi) for each window.

Nucleotide diversity (Pi) was further evaluated using a sliding window analysis to identify regions of high sequence variability. The Pi values ranged from 0 to 0.0727, with a mean of 0.0200. Ten highly variable regions were identified, including *rps16, rps16-trnQ(UUG), trnS(GCU)-trnG(UCC), trnG(UCC), trnY(GUA), trnD(GUC), rpl32, rps15, clpP*, and *ycf1*. The highest Pi value (0.0727) was detected in the *ycf1* region. Nucleotide diversity and detailed information on the highly variable regions are provided in **Supplementary Table 4**. Most of the highly variable regions were located within genic regions, whereas only two intergenic regions – *rps16-trnQ(UUG)* and *trnS(GCU)-trnG(UCC)* exhibited high variability. The majority of variable regions were distributed in the LSC region (*rps16, rps16-trnQ(UUG), trnS(GCU)-trnG(UCC), trnG(UCC), trnY(GUA), trnD(GUC)*, and *clpP*), while three regions (*rpl32, rps15*, and *ycf1*) were located in the SSC region (**Figure 6**).

### 3.4 Simple sequence repeats identification

**Figure 7:**
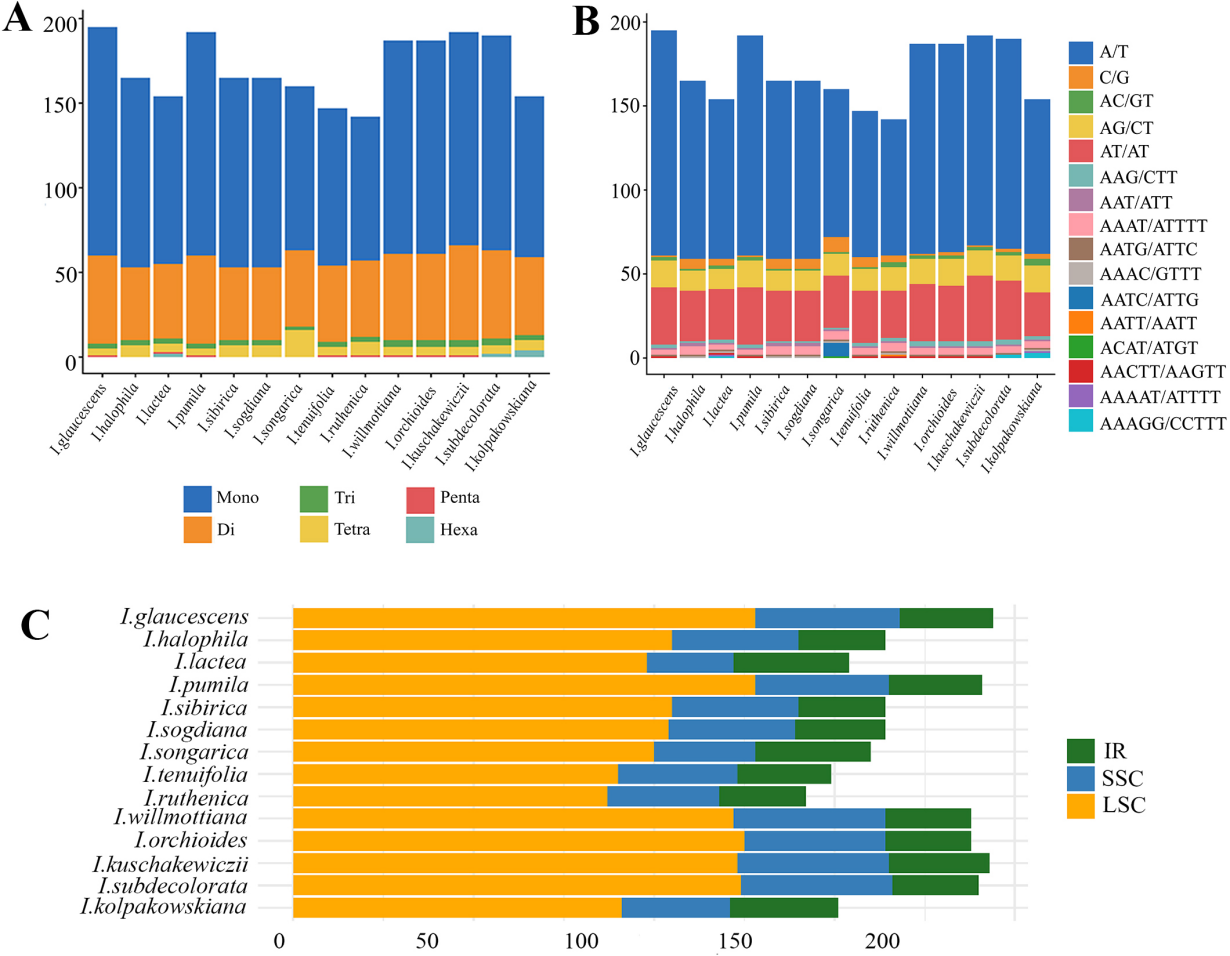
Simple sequence repeats (SSRs) in 14 *Iris* plastomes. **(A)** Number of different repeat types. **(B)** Frequency of identified SSR motifs. **(C)** Regions of repeats in chloroplast genomes.

Plastomes of 14 *Iris* species exhibited considerable variation in SSR content, with *I. glaucescens* containing the highest number of SSRs (195), while *I. ruthenica* had the lowest number (142). In total, 2, 401 microsatellite repeats were identified across the nucleotide sequences of the 14 *Iris* plastomes, with an average of 171.5 SSRs per species. Six types of SSRs were classified based on nucleotide composition: mononucleotide (mono), dinucleotide (di), trinucleotide (tri), tetranucleotide (tetra), pentanucleotide (penta), and hexanucleotide (hexa). Among these, mononucleotide repeats were the most abundant, accounting for 1, 577 SSRs (65.7%), followed by dinucleotide repeats (668; 27.8%), tetranucleotide repeats (89; 3.7%), trinucleotide repeats (45; 1.9%), pentanucleotide repeats (14; 0.6%), and hexanucleotide repeats (8; 0.3%) (**Figure 7A**). In terms of motif composition, mononucleotide (A/T) and dinucleotide (AT/AT) repeats were the most prevalent, representing 1, 525 (63.5%) and 445 (18.5%) of the total SSRs, respectively. Notably, A/T motif dominated the mononucleotide category, accounting for 1, 525 (96.7%) of all mononucleotide repeats (**Figure 7B**). Regarding their distribution within the plastome, SSRs were predominantly located in the LSC region, followed by the SSC and IR regions (**Figure 7C**). Detailed information on the identified SSRs was given in **Supplementary Table 5**.

### 3.5 Codon usage

**Figure 8:**
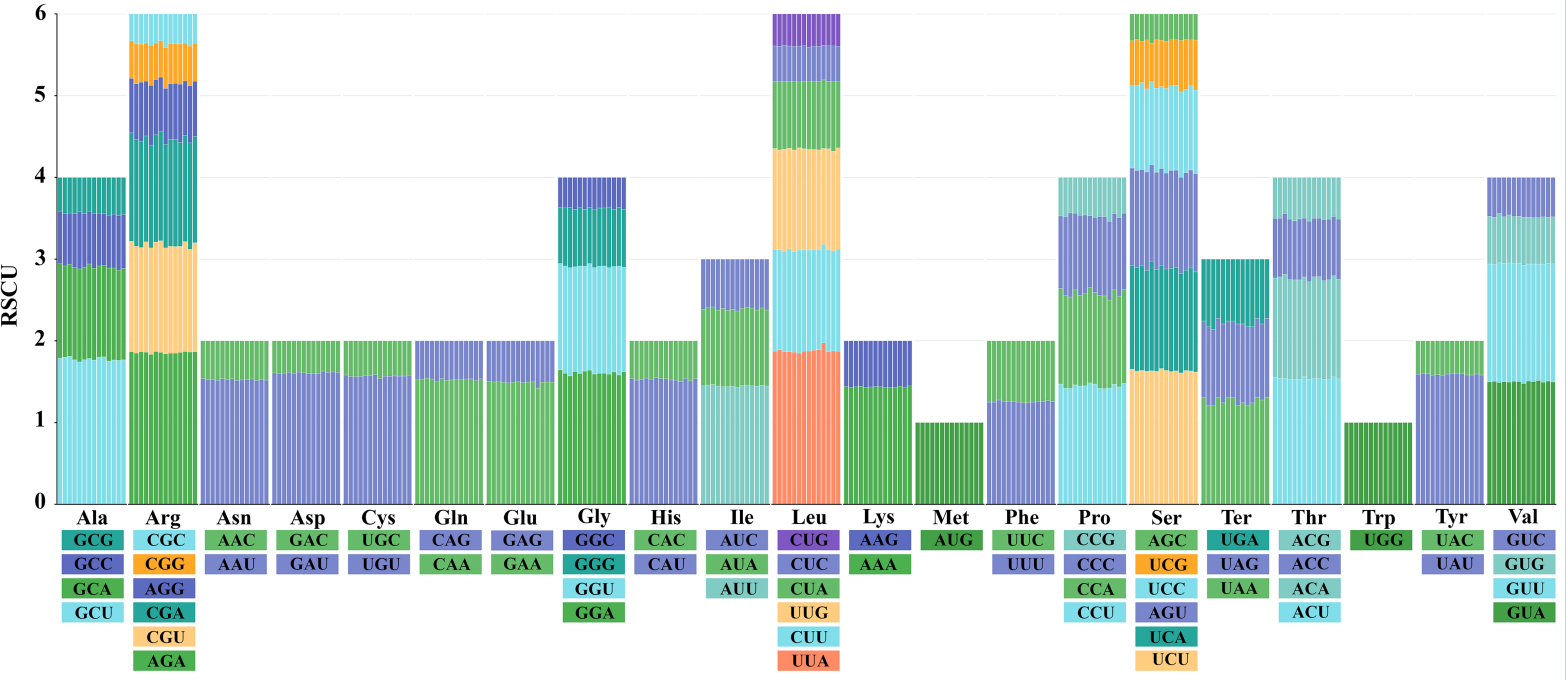
Relative synonymous codon usage (RSCU) values for all protein-coding genes in 14 *Iris* plastomes. Each column within an amino acid group represents one species. Bar height indicates RSCU value; bars are color-coded by synonymous codon as shown in the legend table below. AUG (Met) and UGG (Trp) are shown with RSCU = 1.0 as they have no synonymous codons. Ter, termination (stop) codons.

RSCU values were calculated for all 64 codons across the 14 *Iris* plastomes to determine nonuniform synonymous codon usage. The total number comprised 64 codons, of which 31 were preferred (RSCU > 1.0), 31 were unpreferred low-frequency (RSCU < 1.0), and 2 codons, AUG (Met) and UGG (Trp), showed no bias as they are the only codons for their respective amino acids. Precisely, our findings revealed that *I. kolpakowskiana* exhibited 32 preferred codons, suggesting a slightly broader synonymous codon usage. RSCU values across all species ranged from 0.31 to 1.97, indicating moderate but consistent codon usage bias throughout the studied species of genus (**Figure 8**).

The three most strongly preferred codons were consistent across all 14 species, with UUA (Leu), AGA (Arg), and GCU (Ala) ranking as the top three in nearly every genome. Notably, *I. songarica* showed the highest RSCU value for UUA among all species (1.97), significantly higher than the remaining 13 genomes, which ranged from 1.85 to 1.89, suggesting an elevated preference for this leucine codon in this species. For stop codon usage, UAA was most preferred in *I. glaucescens, I. kuschakewiczii, I. orchioides, I. pumila, I. subdecolorata*, and *I. willmottiana* (1.31), while the lowest value was recorded in *I. halophila, I. kolpakowskiana, I. ruthenica*, and *I. sogdiana* (1.21). UGA showed its highest usage in *I. kolpakowskiana* (0.86) and its lowest in *I. kuschakewiczii, I. subdecolorata*, and *I. willmottiana* (0.72). For UCG (Ser), *I. songarica* displayed the highest value (0.63) and *I. lactea* the lowest (0.47), indicating differences based on species in serine codon selection (**Supplementary Table 6**).

### 3.6 Phylogenetic analysis

**Figure 9:**
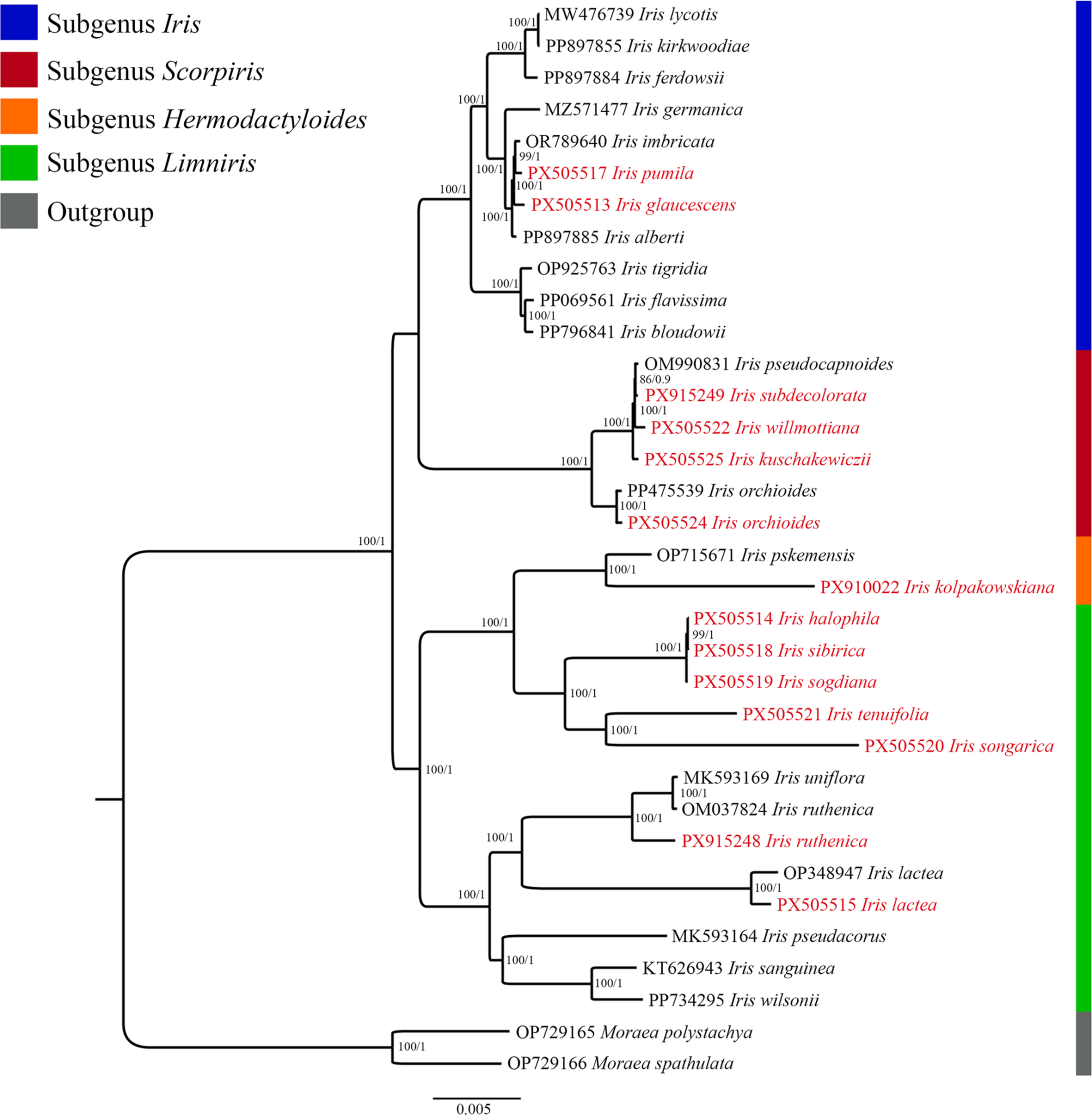
Maximum likelihood (ML) and Bayesian inference (BI) phylogenetic tree of *Iris* species based on complete plastome nucleotide sequences. Nodal support values are indicated as ML bootstrap values/BI posterior probabilities. Subgenera are color-coded as follows: *Iris* (blue), *Scorpiris* (red), *Hermodactyloides* (orange), and *Limniris* (green). Outgroup taxa (*Moraea* species) are shown in gray. Species analyzed in this study are highlighted in red font.

The phylogenetic relationships among *Iris* species (32 ingroup samples, including 14 sequenced in this study and 18 from NCBI GenBank, and 2 outgroup samples), inferred from complete plastome sequences using ML, BI (**Figure 9**), and NJ (**Supplementary Figure 1**) analyses, yielded highly congruent tree topologies with strong statistical support across most nodes. The genus *Iris* formed a well-supported monophyletic clade, clearly separated from the outgroup taxa (*M. polystachya* and *M. spathulata*). Within *Iris*, species were grouped into three major clades corresponding to the subgenera *Iris*, *Scorpiris*, *Limniris*, and *Hermodactyloides*, each receiving strong support from bootstrap and posterior probability analyses. The representatives of subgenus *Hermodactyloides* shared one clade with species from subgenus *Limniris*. Notably, the species newly sequenced in this study were consistently placed within their respective subgenera, confirming their taxonomic assignments. High support values at most nodes indicate strong resolution of phylogenetic relationships among the studied taxa. Overall, the plastome-based phylogeny provides support for the placement of the sampled species within major *Iris* lineages. However, these results should be interpreted as reflecting plastid genome history rather than a fully resolved species-level or subgeneric phylogeny.

### 3.7 Molecular dating and biogeography

**Figure 10:**
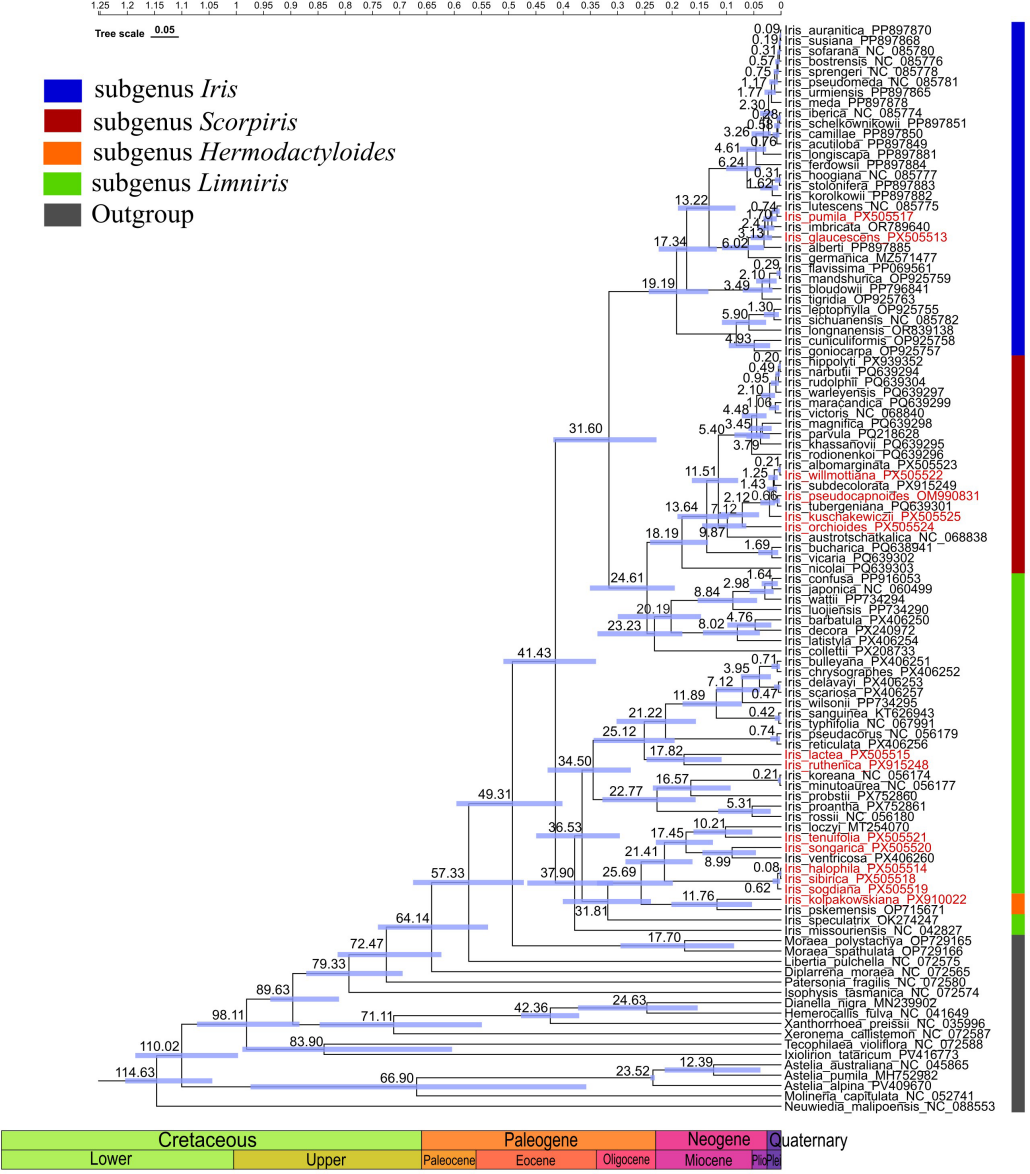
Divergence time estimates of *Iris* inferred from 65 CDS loci using MCMCtree. Blue node bars represent 95% credibility intervals, and numbers at the nodes indicate mean divergence times (Ma). The samples labeled in red denote species sequenced in this study. Colors correspond to the subgeneric classification indicated in the upper-left legend. The geological time scale is shown at the bottom of the figure.

The genus *Iris* likely originated approximately 49.31 Ma (95% CI: 40.13–59.60 Ma), based on 65 CDS loci (**Figure 10**). Diversification within the crown group of *Iris* started around 41.43 Ma (95% CI: 33.97–50.97 Ma). The crown ages of subgenus *Scorpiris* and subgenus *Iris* were estimated at 18.19 Ma (95% CI: 13.41–24.06 Ma) and 19.19 Ma (95% CI: 13.33–24.29 Ma), respectively. One *Limniris* lineage, associated with subgenus *Scorpiris*, began to diversify at approximately 23.23 Ma (95% CI: 18.13–33.71 Ma), whereas another *Limniris* lineage, associated with subgenus *Hermodactyloides*, diverged around 37.90 Ma (95% CI: 30.84–46.58 Ma). The stem age of subgenus *Hermodactyloides* was estimated at 25.69 Ma (95% CI: 19.87–33.78 Ma).

**Figure 11:**
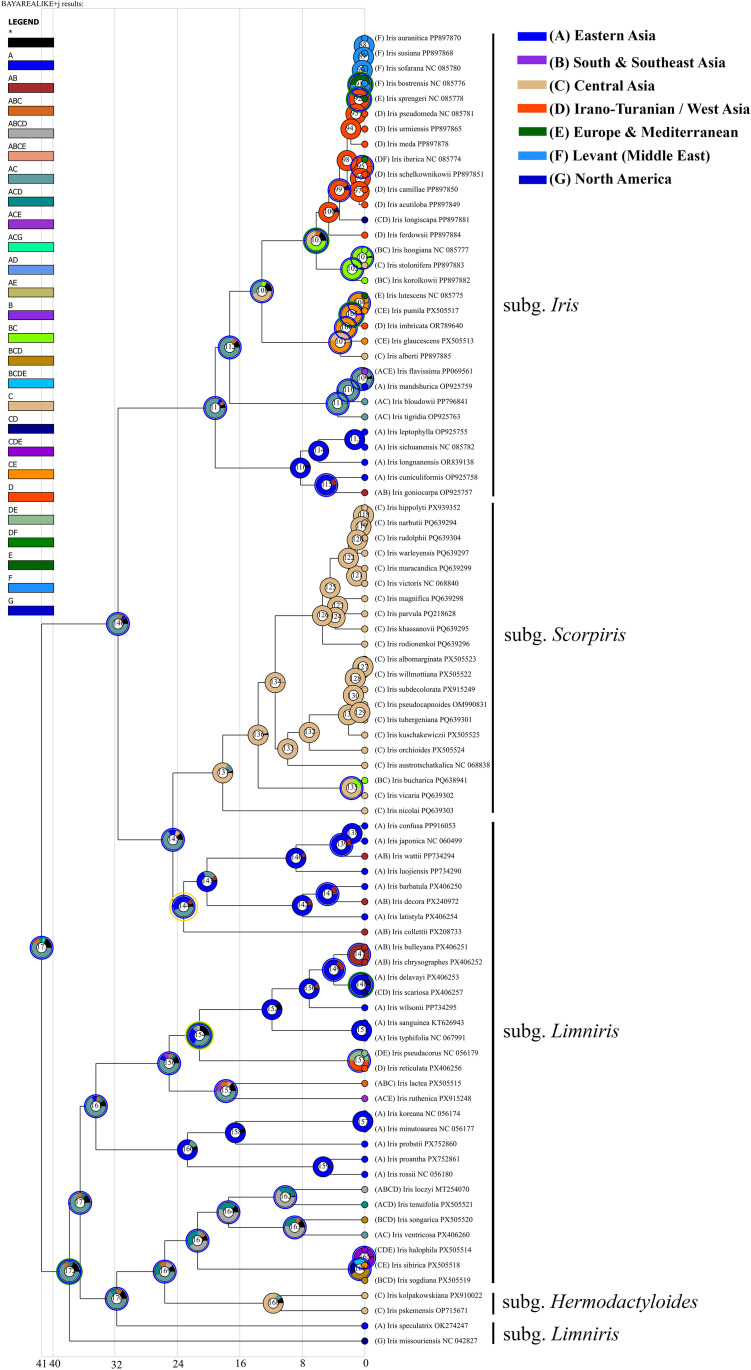
Biogeographic reconstruction of *Iris* based on the BAYAREALIKE+J model, identified as the best-supported by BioGeoBEARS in RASP based on the highest AICwt. Colored symbols at the nodes indicate inferred ancestral ranges. The legend on the left shows all reconstructed combinations of ancestral areas, whereas the panel on the right defines the corresponding geographic regions: **(A)** Eastern Asia, **(B)** South and Southeast Asia, **(C)** Central Asia, **(D)** Irano-Turanian region (West Asia), **(E)** Europe and the Mediterranean, **(F)** Levant (Middle East), and **(G)** North America.

BioGeoBEARS model comparison supported BAYAREALIKE+J as the best-fitting model, as indicated by the lowest AICc value and the highest Akaike weight (**Supplementary Table 7**). The ancestral-area reconstruction suggested that *Iris* originated in a combined Eastern Asia-Central Asia range (AC) (**Figure 11**). This range was also inferred for several deep internal nodes, suggesting that Eastern and Central Asia (AC) were central to the genus’s early evolutionary history. Within subgenus *Iris*, dispersal was inferred from the ancestral Asian (AC) range into the Irano-Turanian region (West Asia) (D). Subgenus *Scorpiris* was reconstructed as having originated in Central Asia (C). In one *Limniris* lineage, dispersal occurred from Eastern Asia (A) to South Asia (B). Within the clade comprising subgenus *Limniris* and subgenus *Hemerodactyloides*, a long-distance dispersal event gave rise to *I. missouriensis* in North America (G).

## 4 Discussion

### 4.1 General characteristics and structure of Iris plastomes

In this study, complete plastomes of 14 *Iris* species collected in Kazakhstan were sequenced using Illumina high-throughput sequencing technology. Genome assembly and annotation revealed that all plastomes exhibited the typical quadripartite structure, consisting of LSC region, SSC region, and two IR regions (**Figure 3**). The overall AT content was higher than the GC content, which is consistent with patterns commonly observed in other angiosperm plastomes (Almerekova et al., 2024; Kim et al., 2025). The total plastome size among the studied species ranged from 150, 612 bp to 155, 046 bp, while the GC content varied from 37.8% to 38.9%. These values are in agreement with previously reported plastome characteristics in *Iris* species (Feng et al., 2022; Choi and Lee, 2024). A total of 133 genes were annotated across the 14 plastomes, including 87 PCG, 38 tRNA genes, and 8 rRNA genes (**Table 1**), indicating a high level of similarity among the plastomes. These gene counts are consistent with previously published *Iris* plastomes (Liu et al., 2020; Cai et al., 2021; Feng et al., 2022).

Although IR regions are generally conserved, their expansion and contraction are common phenomena that contribute significantly to variation in plastome size and structural rearrangements (Wicke et al., 2011). Comparative analysis of the 14 *Iris* species showed that the IRa/SSC and IRb/SSC junctions were located within the *ycf1* gene (**Figure 4**), a pattern widely observed across many plant taxa (Huang et al., 2022b; Oyuntsetseg et al., 2024). However, in *I. kolpakowskiana*, the duplicated copy of the *ycf1* gene was absent at the IRb/SSC junction and instead located entirely within the IRb region. Similar patterns have been reported in other *Iris* plastomes (Feng et al., 2022; Choi and Lee, 2024). Overall, the IR boundary regions exhibited a high degree of structural conservation across all 14 *Iris* plastomes. Minor expansions and contractions of the IR regions were detected, reflected by small shifts in gene positions and intergenic distances, further highlighting the dynamic yet conserved nature of plastome architecture within the genus. Consistent with this observation, mVISTA-based comparisons demonstrated that IR regions are more conserved than the LSC and SSC regions (**Figure 5**).

Taken together, the gene content and plastome structure of the 14 *Iris* species show a high degree of conservation, suggesting evolutionary stability of plastid genomes within the genus *Iris*.

### 4.2 Identification of molecular markers

Nucleotide diversity (Pi) analysis is an approach for identifying highly variable (hotspot) regions within plastomes. These regions are particularly useful as potential DNA barcodes for species identification (Park et al., 2019; Lin et al., 2023) and as informative markers in phylogenetic analyses (Dong et al., 2015; Li et al., 2021; Jiang et al., 2022; Almerekova et al., 2024). In the present study, most of the identified hotspot regions were located in LSC and SSC regions, whereas the IR regions exhibited a high level of conservation. This pattern is consistent with observations reported across numerous plant lineages (He et al., 2020; Xiao et al., 2025) and is generally attributed to lower mutation rates in IR regions relative to higher evolutionary rates in single-copy regions (Ahmed and Rahman, 2024). A total of ten highly variable regions were identified (**Figure 6**), comprising eight genic regions (*rps16, trnG(UCC), trnY(GUA), trnD(GUC), rpl32, rps15, clpP*, and *ycf1*) and two intergenic spacers (*rps16–trnQ(UUG)* and *trnS(GCU)–trnG(UCC)*). Previous studies on *Iris* plastomes (Ortikov et al., 2023; Choi and Lee, 2024) have similarly reported elevated polymorphism in several of these regions, particularly *rps16–trnQ(UUG)*, *rpl32*, *rps15*, *clpP*, and *ycf1*. Notably, many of the polymorphic loci identified here have been widely employed as molecular markers in phylogenetic studies of *Iris* species (Guo and Wilson, 2013; Wilson, 2017; Boltenkov and Artyukova, 2024), further supporting their utility for evolutionary and taxonomic investigations.

### 4.3 Analysis of simple sequence repeats

SSRs are widely distributed in plastomes and are important molecular markers extensively used in population and conservation genetics (Yermagambetova et al., 2023; Ye et al., 2024; Moosavi et al., 2025). In this study, six types of SSRs were identified, with a clear predominance of mononucleotide repeats. Notably, all six SSR types were detected in only five plastomes (**Figure 7**). The dominance of mononucleotide repeats, particularly A/T motifs (65.7% of total SSRs), is consistent with patterns reported for most angiosperm plastomes, where A/T-rich SSRs prevail due to the intrinsic AT bias of plastid genomes (Kuang et al., 2011; George et al., 2015). The much lower frequency of longer repeat motifs (tri-, tetra-, penta-, and hexanucleotides) likely reflects structural constraints and the relatively conserved nature of plastomes compared to nuclear genomes (Raubeson et al., 2007; George et al., 2015). In this study, SSRs were unevenly distributed across plastome regions, with the highest density observed in the LSC region, followed by the SSC region, and the lowest in the IR regions. The predominance of A/T-rich mononucleotide repeats and the higher abundance of SSRs in the LSC region observed here are consistent with SSR patterns reported in other *Iris* plastomes (Ortikov et al., 2023; Choi and Lee, 2024). In contrast, the lower SSR abundance in IR regions may be associated with their higher sequence conservation, copy-dependent repair mechanisms, and the presence of essential genes, such as rRNA genes, which may constrain mutation accumulation (Feng et al., 2022; Javaid et al., 2023; Tao et al., 2024). The relatively high number of SSRs observed in *I. glaucescens* and the lower number in *I. ruthenica* may reflect lineage-specific evolutionary dynamics, including differences in mutation rates, genome stability, or demographic history (Zhu et al., 2021).

The SSR dataset generated in this study provides a useful foundation for developing plastid markers for future population-level and conservation-oriented studies of *Iris*. Because plastid SSRs are generally uniparentally inherited and relatively conserved compared with nuclear SSRs, they may be particularly useful for tracing maternal lineages, assessing plastid haplotype diversity, and supporting species identification in taxonomically complex groups (Provan et al., 2001; Feng et al., 2022; Park et al., 2019). These markers may also help evaluate population differentiation and guide the selection of genetically representative populations for conservation, especially in rare and endemic *Iris* species (Choi and Lee, 2024). In addition, plastome SSRs may contribute to germplasm characterization and breeding-related studies by providing markers for genetic resource management (Lee et al., 2019; Jayaswall et al., 2023). Overall, the SSR patterns identified here represent valuable molecular resources for population genetic analyses, conservation genetics, species identification, phylogeographic investigations, and future breeding studies within the genus Iris.

### 4.4 Сodon usage

Codon usage bias is an important genomic feature reflecting the combined effects of mutational pressure and natural selection on coding sequences (Shen et al., 2024; Jia et al., 2025). The analysis of RSCU across 14 *Iris* species (**Figure 8**) revealed a generally conserved codon usage pattern, with most codons exhibiting similar RSCU values among taxa, indicating a high degree of evolutionary conservation in plastome coding sequences.

The majority of preferred codons (RSCU > 1) end with A or U, whereas codons ending with G or C are generally underrepresented (RSCU < 1). This trend is particularly evident for amino acids with multiple synonymous codons, such as leucine, serine, and arginine, where A/U-ending codons are consistently favored over their G/C-ending counterparts. Although minor interspecific variation is observed in some codons, especially among highly degenerate amino acids, these differences are relatively small and do not alter the overall pattern of codon preference.

The observed bias toward A/U-ending codons is a characteristic feature of plastid genomes and reflects the underlying AT-rich nucleotide composition of angiosperm plastomes. Similar codon usage patterns have been widely reported in other monocot plastid genomes (Feng et al., 2022; Wang et al., 2022; Jia et al., 2025; Wu et al., 2025), suggesting that mutational bias toward A/T nucleotides plays a dominant role in shaping synonymous codon usage. The overall conservation of RSCU values among *Iris* species further indicates that plastome evolution within the genus is relatively stable, with limited divergence in coding sequence composition.

### 4.5 Phylogenetic relationships within Iris

Plastome sequences are widely recognized as an effective tool for resolving phylogenetic relationships (Peng et al., 2023; Song et al., 2024b). In particular, complete plastome nucleotide data provide greater phylogenetic resolution and support than analyses based on single or a few gene regions (Shen et al., 2024; Jia et al., 2025; Yermagambetova et al., 2025), owing to their larger dataset size and reduced stochastic error. Phylogenetic analyses based on complete plastome sequences, using both ML and BI approaches, produced highly congruent topologies with strong statistical support, underscoring the robustness and reliability of plastome-scale data. The recovery of *Iris* as a well-supported monophyletic group, clearly separated from the outgroup taxa (*M. polystachya* and *M. spathulata*), is consistent with previous phylogenetic studies of Iridaceae and supports the genus’s distinct evolutionary lineage (Wilson, 2011). However, the subgenus *Limniris* was recovered as paraphyletic, with taxa of the subgenus *Hermodactyloides* nested within the *Limniris* clade with strong statistical support. This pattern indicates that current subgeneric delimitations do not accurately reflect evolutionary relationships within the genus *Iris*. Previous molecular phylogenetic studies have likewise demonstrated that subgenus *Limniris* is not monophyletic, being recovered as either paraphyletic or polyphyletic depending on taxon sampling and molecular markers (Wilson, 2009; Wilson, 2011; Raycheva et al., 2011; Meerow et al., 2017; Jiang et al., 2018; Choi et al., 2020; Crespo et al., 2024). Our plastome-based phylogeny is congruent with these findings and provides further evidence that *Limniris*, as currently circumscribed, represents an artificial grouping, with *Hermodactyloides* embedded within its evolutionary lineage.

Nevertheless, the plastome-based phylogeny should be interpreted with caution. Although complete plastome sequences provide substantial phylogenetic signals, chloroplast genomes represent a single, usually uniparentally inherited and largely non-recombining locus. Therefore, plastid phylogenies may not fully reflect species-level evolutionary history, particularly in taxonomically complex groups such as *Iris*, where hybridization, introgression, chloroplast capture, and incomplete lineage sorting may occur (Arnold et al., 2012; Hamlin and Arnold, 2014; Lian et al., 2016). These processes can generate cytonuclear discordance and lead to incongruence between plastid- and nuclear-based phylogenetic reconstructions (Dong et al., 2022). Accordingly, the phylogenetic tree presented here should be regarded primarily as reflecting plastid genome evolution rather than a fully resolved species tree for the genus. Future studies incorporating nuclear genomic data, multiple accessions per species, and broader taxon sampling will be necessary to test the robustness of the inferred relationships and to clarify species-level and subgeneric relationships within *Iris*.

### 4.6 Divergence time estimation and biogeographical patterns

The estimated stem age of *Iris* in this study was 49.31 Ma (95% CI: 40.13–59.60 Ma), based on CDS data (**Figure 10**). This estimate is older than that reported by Joyce et al. (2018), who used BEAST and four plastid loci in a family-wide analysis calibrated with *Geosiris* and estimated the divergence of *Iris* at approximately 37.7 Ma. However, the stem age estimated here is broadly consistent with that of Goldblatt et al. (2008), who inferred that *Iris* diverged from *Moraea* around 45 Ma using five plastid regions. Previously published divergence time estimates for *Iris* have been based on plastid data (Goldblatt et al., 2008; Joyce et al., 2018). In particular, plastid genome and nuclear datasets may yield different age estimates (Dong et al., 2022). Still, the limited number of studies on divergence time estimation in *Iris* makes it difficult to compare results. In addition, because of the dataset’s size and associated computational demands, divergence-time analyses were conducted using MCMCtree, which employs an approximate-likelihood method to estimate node ages efficiently. This approach has been reported to yield somewhat older age estimates under certain conditions, as discussed by Barba-Montoya et al (Barba-Montoya et al., 2017). Differences in taxon sampling, molecular data, analytical methods, and calibration points may all contribute to variation in divergence time estimates (Soares and Schrago, 2012). Therefore, further dated phylogenetic studies based on nuclear genomic data would improve our understanding of the timing of diversification in *Iris*.

Biogeographic reconstruction in the present study suggests that *Iris* originated within a broad ancestral range spanning Eastern Asia and Central Asia (**Figure 11**). This result is generally consistent with earlier hypotheses suggesting an Asian origin of the genus (Rodionenko, 1987). To date, few studies have specifically focused on the biogeographic origin of *Iris*. One relevant broader-scale study is that of Joyce et al. (2018), who reconstructed the historical biogeography of Iridaceae and suggested that *Iris* diverged from its African sister lineages within a broad ancestral range including Eurasia, Africa, and North America. At lower taxonomic levels, our inference of a Central Asian origin for section *Oncocyclus* of subgenus *Iris* is consistent with the findings of Volis et al. (2024). Similarly, the recent study of Salimbahrami et al. (2026), which suggested a Pamir-Alay origin and subsequent diversification of subgenus *Scorpiris* during the Early Miocene, is broadly congruent with our results.

The inferred importance of Central Asia in the early diversification of *Iris* may also be interpreted in the context of major Cenozoic geological and paleoclimatic changes across Eurasia (Wang et al., 2016). The uplift of the Pamir-Tian Shan Mountain systems, retreat of the proto-Paratethys Sea, reduced moisture transport into inland Asia, and progressive aridification likely promoted habitat fragmentation and the formation of heterogeneous montane, steppe, and semi-arid environments (Bosboom et al., 2019; Caves Rugenstein and Chamberlain, 2018). Similar links between mountain uplift, aridification, habitat heterogeneity, and lineage diversification have been suggested for other Central Asian and Irano-Turanian plant groups, including *Lagochilus* (Zhang et al., 2017), *Acantholimon* (Manafzadeh et al., 2019), *Rhaponticoides* (Bozkurt et al., 2022), and *Iris* subgenus *Scorpiris* (Salimbahrami et al., 2026). These environmental changes may have created ecological opportunities for diversification and range shifts in Iris lineages, particularly in Central Asian and Irano-Turanian regions. However, because these links are based mainly on temporal and geographic correspondence rather than direct testing, they should be regarded as hypotheses requiring further evaluation using broader taxon sampling, nuclear genomic data, and explicit paleogeographic and paleoclimatic modeling.

The biogeographic interpretations should be considered preliminary. Although our analysis included 88 species representing four subgenera, *Iris* comprises more than 300 accepted species worldwide (Plants of the World Online), and taxon sampling therefore remains incomplete. Limited sampling at both the species and population levels may influence the inferred phylogenetic topology, divergence time estimates, and ancestral area reconstruction. Future studies incorporating multiple accessions per species, broader taxon coverage, and nuclear genomic data will therefore be important for testing the robustness of the phylogenetic and biogeographic patterns inferred here.

An additional methodological consideration concerns the biogeographic model used to reconstruct ancestral areas. Although BAYAREALIKE+J provided the best statistical fit in our analysis, interpretations based on “+J” models should be treated cautiously. The “+J” parameter was introduced to model founder-event speciation and may improve model fit in some empirical datasets, particularly those involving long-distance dispersal or island clades (Matzke, 2014). However, the use of “+J” models in BioGeoBEARS has been debated because founder-event speciation may strongly influence ancestral range estimates and increase the inferred importance of jump dispersal events (Ree and Sanmartín, 2018). Subsequent work has argued that comparisons between +J and non-+J models can be statistically valid under related likelihood frameworks (Matzke, 2022). Therefore, the biogeographic patterns inferred here should be regarded as model-based hypotheses rather than definitive reconstructions of the historical biogeography of *Iris*.

## 5 Conclusion

In the present study, plastome nucleotide sequences of 14 *Iris* species were analyzed to investigate comparative genomics, molecular dating, and biogeography. Several highly variable regions (*rps16, rps16–trnQ(UUG), trnS(GCU)–trnG(UCC), trnG(UCC), trnY(GUA), trnD(GUC), rpl32, rps15, clpP*, and *ycf1*) were identified and may serve as useful markers for phylogenetic studies within the genus. In addition, the detected microsatellites (SSRs) represent valuable resources for population genetic analyses, particularly for rare and endemic species, and may contribute to the development of effective conservation strategies. The phylogenetic analysis revealed that the genus *Iris* formed a well-supported monophyletic clade, clearly separated from the outgroup taxa. However, the subgenus *Limniris* was recovered as paraphyletic. Due to the limited availability of comparable data, the results of molecular dating and biogeographic analyses remain preliminary. Nevertheless, our findings suggest that the stem age of *Iris* is approximately 49.31 Ma and that the genus likely originated across a broad ancestral range spanning Eastern Asia and Central Asia. Overall, this study provides a foundation for future phylogenomic, divergence time, and biogeographic research on *Iris*. Further investigations incorporating both plastome and nuclear genomic data, along with expanded taxon sampling, are necessary to achieve a more comprehensive understanding of the evolutionary history and diversification of this complex genus.

## Data Availability

The sequences obtained in this study were deposited in the NCBI GenBank under accession numbers PX505513-PX505515, PX505517-PX505522, PX505524-PX505525, PX915248-PX915249, and PX910022. The raw data are available under BioProject PRJNA1471469.

## References

[B1] Ahmed S. S. Rahman M. O. (2024). Deciphering the complete chloroplast genome sequence of Meconopsis torquata Prain: Insights into genome structure, comparative analysis and phylogenetic relationship. Heliyon 10, e36204. doi: 10.1016/j.heliyon.2024.e36204 39224270 PMC11367419

[B2] Aitkenova A. A. Atazhanova G. A. Badekova K. Z. Samorodov A. V. Akhmetova S. B. Ishmuratova M. Y. . (2025). Assessment of the acute toxicity of ethanol extract from the rhizomes of Iris scariosa L. in mice. Front. Pharmacol. 16, 1701980. doi: 10.3389/fphar.2025.1701980 41601980 PMC12832804

[B3] Alexeeva N. B. (2018). A taxonomic revision of Iris section Psammiris (Iridaceae) in Russia. Phytotaxa 340, 201–216. doi: 10.11646/phytotaxa.340.3.1

[B4] Alikhanova A. Osmonali B. Turuspekov Y. Almerekova S. (2025). The distribution features of the rare species Iris Kuschakewiczii B. Fedtsch. on the territory of Kazakhstan. Exp. Biol. 2, 4–16. doi: 10.26577/bb202510321

[B5] Almerekova S. Yermagambetova M. Ivaschenko A. Turuspekov Y. Abugalieva S. (2024). Comparative analysis of plastome sequences of seven Tulipa L. (Liliaceae Juss.) species from section Kolpakowskianae Raamsd. Ex Zonn and Veldk. Int. J. Mol. Sci. 25, 7874. doi: 10.3390/ijms25147874 39063115 PMC11277319

[B6] Apostol M. Draghia L. Sîrbu C. Efrose R. C. Flemetakis E. Hlihor R. M. . (2024). Morphological, anatomical, physiological and genetic studies of iris Aphylla L. wild species conservation in “ex situ” conditions. Agriculture 14, 2358. doi: 10.3390/agriculture14122358 30654563

[B7] Arnold M. L. Ballerini E. S. Brothers A. N. (2012). Hybrid fitness, adaptation and evolutionary diversification: lessons learned from Louisiana Irises. Heredity 108, 159–166. doi: 10.1038/hdy.2011.65 21792222 PMC3282389

[B101] Baitulin I. O. (2014). Red Data Book of Kazakhstan, 2nd ed. rev. and suppl Vol. 2 (Astana: LTD “ArtPrintXXI”). Plants.

[B8] Bankevich A. Nurk S. Antipov D. Gurevich A. A. Dvorkin M. Kulikov A. S. . (2012). SPAdes: A new genome assembly algorithm and its applications to single-cell sequencing. J. Comput. Biol. 19, 455–477. doi: 10.1089/cmb.2012.0021 22506599 PMC3342519

[B9] Barba-Montoya J. dos Reis M. Yang Z. (2017). Comparison of different strategies for using fossil calibrations to generate the time prior in Bayesian molecular clock dating. Mol. Phylogenet. Evol. 114, 386–400. doi: 10.1016/j.ympev.2017.07.005 28709986 PMC5546266

[B10] Benoit-Vical F. Imbert C. Bonfils J. P. Sauvaire Y. (2003). Antiplasmodial and antifungal activities of iridal, a plant triterpenoid. Phytochemistry 62, 747–751. doi: 10.1016/s0031-9422(02)00625-8 12620327

[B11] Bensari S. (2020). Phytochemical profiles of Iris unguicularis Poir. with antioxidant, antibacterial, and anti-Alzheimer activities. Acta Nat. Sci. 7, 74–87. doi: 10.2478/asn-2020-0021

[B12] Bo W. Wang S. Xing G. Wang Y. (2025). Genetic diversity analysis of Iris germanica cultivars based on ISSR and SRAP molecular markers. Front. Plant Sci. 16, 1629234. doi: 10.3390/f15081377 41001109 PMC12457380

[B13] Bolger A. M. Lohse M. Usadel B. (2014). Trimmomatic: A flexible trimmer for illumina sequence data. Bioinformatics 30, 2114–2120. doi: 10.1093/bioinformatics/btu170 24695404 PMC4103590

[B14] Boltenkov E. V. Artyukova E. V. (2024). Updated Taxonomy of Iris scariosa (Iridaceae) Inferred from Morphological and Chloroplast DNA Sequence Data with Remarks on Classification of Iris subg. Iris. Plants 13, 2349. doi: 10.3390/plants13172349 39273833 PMC11397725

[B15] Borowiec M. L. (2016). AMAS: a fast tool for alignment manipulation and computing of summary statistics. PeerJ 4, e1660. doi: 10.7717/peerj.1660 26835189 PMC4734057

[B16] Bosboom R. E. Dupont-Nivet G. Grothe A. Brinkhuis H. Villa G. Mandic O. . (2019). Paleogene evolution and demise of the proto-Paratethys Sea in Central Asia: role of intensified tectonic activity at ca. 41 Ma? Basin Res. 31, 461–486. doi: 10.1111/bre.12330 40046247

[B17] Bozkurt M. Calleja Alarcón J. A. Uysal T. García-Jacas N. Ertuğrul K. Susanna A. (2022). Biogeography of Rhaponticoides, an Irano-Turanian element in the Mediterranean flora. Sci. Rep. 12, 21435. doi: 10.1038/s41598-022-24947-3 36539442 PMC9768164

[B18] Cai X. Zhang B. Wang S. Cheng Y. Wang H. (2021). Characterization and phylogenetic analysis of the chloroplast genome of Iris lactea var. chinensis. Mitochondrial DNA Part B 6, 1490–1491. doi: 10.1080/23802359.2020.1847611 33997284 PMC8081313

[B19] Caves Rugenstein J. K. Chamberlain C. P. (2018). The evolution of hydroclimate in Asia over the Cenozoic: A stable-isotope perspective. Earth Sci. Rev. 185, 1129–1156. doi: 10.1016/j.earscirev.2018.09.003 38826717

[B20] Chen M. L. Feng Y. M. Zhang X. Y. Xu F. Kang Q. Q. Ning X. J. . (2024). Phylogenetic study of Iris plants in China based on chloroplast matK gene and nuclear ITS gene. PREPRINT (Version 1), Research Square. doi: 10.21203/rs.3.rs-4992391/v1

[B21] Choi B. Weiss-Schneeweiss H. Temsch E. M. So S. Myeong H. H. Jang T. S. (2020). Genome size and chromosome number evolution in Korean Iris L. species (Iridaceae Juss.). Plants 9, 1284. doi: 10.3390/plants9101284 32998465 PMC7650623

[B22] Choi T. Y. Lee S. R. (2024). Complete plastid genome of Iris orchioides and comparative analysis with 19 Iris plastomes. PloS One 19, e0301346. doi: 10.1371/journal.pone.0301346 38578735 PMC10997070

[B23] Cohen J. I. Turgman-Cohen S. (2023). The conservation genetics of Iris lacustris (Dwarf lake Iris), a great lakes endemic. Plants 12, 2557. doi: 10.3390/plants12132557 37447118 PMC10346457

[B24] Conran J. G. Christophel D. C. Cunningham L. (2003). An Eocene moncotyledon from Nelly Creek, Central Australia, with affinities to Hemerocallidaceae (Lilianae: Asparagales). Alcheringa: An. Australas. J. Palaeontology 27, 107–115. doi: 10.1080/03115510308619551 37339054

[B25] Crespo M. B. Martínez-Azorín M. Mavrodiev E. V. (2015). Can a rainbow consist of a single colour? A new comprehensive generic arrangement of the ‘Iris sensu latissimo’clade (Iridaceae), congruent with morphology and molecular data. Phytotaxa 232, 1–78. doi: 10.11646/phytotaxa.232.1.1

[B26] Crespo M. B. Martínez-Azorín M. Mavrodiev E. V. (2024). Reticulata irises”: a nomenclatural and taxonomic synopsis of the genera Alatavia and Iridodictyum (Iris subg. Hermodactyloides auct. Iridaceae). Plant Biosyst. - Int. J. Dealing Aspects Plant Biosyst. 158, 763–787. doi: 10.1080/11263504.2024.2357304 37339054

[B27] Crișan I. (2025). The genus iris tourn. Ex L.: updates on botany, cultivation, novel niches and impactful applications. Plants 14, 2870. doi: 10.3390/plants14182870 41012022 PMC12473546

[B28] Daniell H. Lin C. S. Yu M. Chang W. J. (2016). Chloroplast genomes: diversity, evolution, and applications in genetic engineering. Genome Biol. 17, 134. doi: 10.1186/s13059-016-1004-2 27339192 PMC4918201

[B30] Dong S. S. Wang Y. L. Xia N. H. Liu Y. Liu M. Lian L. . (2022). Plastid and nuclear phylogenomic incongruences and biogeographic implications of Magnolia sl (Magnoliaceae). J. Syst. Evol. 60, 1–15. doi: 10.1111/jse.12727 40046247

[B29] Dong W. Xu C. Li C. Sun J. Zuo Y. Shi S. . (2015). ycf1, the most promising plastid DNA barcode of land plants. Sci. Rep. 5, 8348. doi: 10.1038/srep08348 25672218 PMC4325322

[B31] Doyle J. J. Doyle J. L. (1987). A rapid DNA isolation procedure for small quantities of fresh leaf tissue. Phytochemical Bull. 19, 11–15.

[B32] Duan N. Deng L. Zhang Y. Shi Y. Liu B. (2022). Comparative and phylogenetic analysis based on chloroplast genome of Heteroplexis (Compositae), a protected rare genus. BMC Plant Biol. 22, 605. doi: 10.1186/s12870-022-04000-1 36550394 PMC9773445

[B33] Duchêne S. Lanfear R. Ho S. Y. (2014). The impact of calibration and clock-model choice on molecular estimates of divergence times. Mol. Phylogenet. Evol. 78, 277–283. doi: 10.1016/j.ympev.2014.05.032 24910154

[B34] Dykes W. R. (1913). The genus iris. Cambridge Univ. Press, 245. doi: 10.5962/bhl.title.116246

[B35] Feng J. L. Wu L. W. Wang Q. Pan Y. J. Li B. L. Lin Y. L. . (2022). Comparison analysis based on complete chloroplast genomes and insights into plastid phylogenomic of four Iris species. BioMed. Res. Int. 2022, 2194021. doi: 10.1155/2022/2194021 35937412 PMC9348943

[B39] Frazer K. A. Pachter L. Poliakov A. Rubin E. M. Dubchak I. (2004). VISTA: computational tools for comparative genomics. Nucleic Acids Res. 32, W273–W279. doi: 10.1093/nar/gkh458 15215394 PMC441596

[B40] George B. Bhatt B. S. Awasthi M. George B. Singh A. K. (2015). Comparative analysis of microsatellites in chloroplast genomes of lower and higher plants. Curr. Genet. 61, 665–677. doi: 10.1007/s00294-015-0495-9 25999216

[B41] Givnish T. Spalink D. Lyon S. Hunter S. Zuluaga A. Doucette A. . (2016). Orchid historical biogeography, diversification, Antarctica and the paradox of orchid dispersal. J. Biogeogr. 43, 1905–1916. doi: 10.1111/jbi.12854 40046247

[B42] Goldblatt P. Rodriguez A. Powell M. P. Davies J. T. Manning J. C. Van der Bank M. . (2008). Iridaceae'out of Australasia'? Phylogeny, biogeography, and divergence time based on plastid DNA sequences. Syst. Bot. 33, 495–508. doi: 10.1600/036364408785679806 40631773

[B43] Greiner S. Lehwark P. Bock R. (2019). OrganellarGenomeDRAW (OGDRAW) version 1.3.1: Expanded toolkit for the graphical visualization of organellar genomes. Nucleic Acids Res. 47, W59–W64. doi: 10.1093/nar/gkz238 30949694 PMC6602502

[B44] Guo J. Wilson C. A. (2013). Molecular phylogeny of crested Iris based on five plastid markers (Iridaceae). Syst. Bot. 38, 987–995. doi: 10.1600/036364413x674724 40631773

[B45] Hai X. Cao T. Liu H. Wang M. Cui H. Tong Z. . (2025). Genetic diversity and population structure in Polygonatum cyrtonema Hua as revealed by simple sequence repeat markers. Genet. Resour. Crop Evol. 72, 57–70. doi: 10.1007/s10722-025-02580-z 30311153

[B46] Hamlin J. A. Arnold M. L. (2014). Determining population structure and hybridization for two iris species. Ecol. Evol. 4, 743–755. doi: 10.1002/ece3.964 24683457 PMC3967900

[B47] He S. Yang Y. Li Z. Wang X. Guo Y. Wu H. (2020). Comparative analysis of four Zantedeschia chloroplast genomes: expansion and contraction of the IR region, phylogenetic analyses and SSR genetic diversity assessment. PeerJ 8, e9132. doi: 10.7717/peerj.9132 32509453 PMC7247528

[B48] Huang X. Coulibaly D. Tan W. Ni Z. Shi T. Li H. . (2022a). The analysis of genetic structure and characteristics of the chloroplast genome in different Japanese apricot germplasm populations. BMC Plant Biol. 22, 354. doi: 10.1186/s12870-022-03731-5 35864441 PMC9306182

[B49] Huang Y. Li J. Yang Z. An W. Xie C. Liu S. . (2022b). Comprehensive analysis of complete chloroplast genome and phylogenetic aspects of ten Ficus species. BMC Plant Biol. 22, 253. doi: 10.1186/s12870-022-03643-4 35606691 PMC9125854

[B50] Javaid N. Ramzan M. Jabeen S. Shah M. N. Danish S. Hirad A. H. (2023). Genomic exploration of Sesuvium sesuvioides: comparative study and phylogenetic analysis within the order Caryophyllales from Cholistan desert, Pakistan. BMC Plant Biol. 23, 658. doi: 10.1186/s12870-023-04670-5 38124056 PMC10731703

[B51] Jayaswall K. Sharma H. Jayaswal D. Sagar R. Bhandawat A. Kumar A. . (2023). Development of chloroplast derived SSR markers for genus Allium and their characterization in the allies for genetic improvement of Alliums. S. Afr. J. Bot. 162, 304–313. doi: 10.1016/j.sajb.2023.09.021 38826717

[B52] Jia X. Wei J. Chen Y. Zeng C. Deng C. Zeng P. . (2025). Codon usage patterns and genomic variation analysis of chloroplast genomes provides new insights into the evolution of Aroideae. Sci. Rep. 15, 4333. doi: 10.1038/s41598-025-88244-5 39910236 PMC11799533

[B54] Jiang S. Chen F. Qin P. Xie H. Peng G. Li Y. . (2022). The specific DNA barcodes based on chloroplast genes for species identification of Theaceae plants. Physiol. Mol. Biol. Plants 28, 837–848. doi: 10.1007/s12298-022-01175-7 35592487 PMC9110604

[B53] Jiang Y. L. Huang Z. Liao J. Q. Song H. X. Luo X. M. Gao S. P. . (2018). Phylogenetic analysis of IRIS L. from China on chloroplast TRNL-F sequences. Biologia 73, 459–466. doi: 10.2478/s11756-018-0063-0

[B55] Joyce E. Crayn D. Lam V. Gerelle W. Graham S. Nauheimer L. (2018). Evolution of Geosiris (Iridaceae): Historical biogeography and plastid-genome evolution in a genus of non-photosynthetic tropical rainforest herbs disjunct across the Indian Ocean. Aust. Syst. Bot. 31, 504–522. doi: 10.1071/SB18028 38477348

[B56] Kang Y. J. Kim S. Lee J. Won H. Nam G. H. Kwak M. (2020). Identification of plastid genomic regions inferring species identity from de novo plastid genome assembly of 14 Korean-native Iris species (Iridaceae). PloS One 15, e0241178. doi: 10.1371/journal.pone.0241178 33104732 PMC7588056

[B57] Kassem M. A. (2025). Comparative analysis of chloroplast genomes across 20 plant species reveals evolutionary patterns in gene content, codon usage, and genome structure. Int. J. Plant Biol. 16, 105. doi: 10.3390/ijpb16030105 30654563

[B8000] Kim Y. Jeong S. Baasanmunkh S. Kim Y. Choi H. J. Park I. (2025). Interactions between chloroplast and mitochondrial genomes in 11 Salix species. Front. Plant Sci. 16, 1693183. doi: 10.3389/fpls.2025.1693183 41358344 PMC12678270

[B37] Komarov V. L. (Ed.) (1935). Flora SSSR. Tom 4: Lileynye, Orkhidnye, Lastovnevye, Grechishnye i dr. [Flora of the USSR. Vol. 4: Liliaceae, Orchidaceae, Asclepiadaceae, Polygonaceae, etc.] (Leningrad: Izdatel'stvo AN SSSR). (In Russian).

[B58] Kuang D. Y. Wu H. Wang Y. L. Gao L. M. Zhang S. Z. Lu L. (2011). Complete chloroplast genome sequence of Magnolia kwangsiensis (Magnoliaceae): implication for DNA barcoding and population genetics. Genome 54, 663–673. doi: 10.1139/g11-026 21793699

[B59] Kumar S. Stecher G. Suleski M. Sanderford M. Sharma S. Tamura K. (2024). MEGA12: Molecular evolutionary genetic analysis version 12 for adaptive and green computing. Mol. Biol. Evol. 41, msae263. doi: 10.1093/molbev/msae263 39708372 PMC11683415

[B60] Lawrence G. H. M. (1953). A reclassification of the genus Iris. Gentes Herbarum 8, 346–371.

[B61] Lee K. J. Lee G.-A. Lee J.-R. Sebastin R. Shin M.-J. Cho G.-T. . (2019). Genetic diversity of sweet potato (Ipomoea batatas L. Lam) germplasms collected worldwide using chloroplast SSR markers. Agronomy 9, 752. doi: 10.3390/agronomy9110752 30654563

[B62] Li H. Wu M. Lai Q. Zhou W. Song C. (2023). Complete chloroplast of four Sanicula taxa (Apiaceae) endemic to China: Lights into genome structure, comparative analysis, and phylogenetic relationships. BMC Plant Biol. 23, 444. doi: 10.1186/s12870-023-04447-w 37730528 PMC10512634

[B63] Li H. Xiao W. Tong T. Li Y. Zhang M. Lin X. . (2021). The specific DNA barcodes based on chloroplast genes for species identification of Orchidaceae plants. Sci. Rep. 11, 1424. doi: 10.1038/s41598-021-81087-w 33446865 PMC7809279

[B64] Lian X. Luo G. Li H. Xu W. Xiao Y. Bi X. (2016). Reciprocal difference of interspecific hybridization between three different colours of Iris dichotoma and I. domestica. J. Hortic. Sci. Biotechnol. 91, 483–490. doi: 10.1080/14620316.2016.1173525 37339054

[B65] Librado P. Rozas J. (2009). DnaSP v5: a software for comprehensive analysis of DNA polymorphism data. Bioinformatics 25, 1451–1452. doi: 10.1093/bioinformatics/btp187 19346325

[B66] Lin X. Lee S. Y. Ni J. Zhang X. Hu X. Zou P. . (2023). Comparative analyses of chloroplast genome provide effective molecular markers for species and cultivar identification in Bougainvillea. Int. J. Mol. Sci. 24, 15138. doi: 10.3390/ijms242015138 37894819 PMC10607086

[B67] Liu Z. Yu X. Cui P. Tian X. (2020). The complete chloroplast genome of Iris tectorum (Iridaceae). Mitochondrial DNA Part B 5, 1561–1562. doi: 10.1080/23802359.2020.1742599 37339054

[B68] Maciunas E. Conran J. Bannister J. Paull R. Lee D. (2011). Miocene Astelia (Asparagales: Asteliaceae) macrofossils from southern New Zealand. Aust. Syst. Bot. 24, 19–31. doi: 10.1071/SB10035 38477348

[B76] Majeed H. O. Faraj J. M. Rasul K. S. Lateef D. D. Tahir N. A. R. (2024). Evaluation of the genetic diversity and population structure of reticulated iris accessions in the Iraqi Kurdistan region using SCoT and SRAP markers. Genet. Resour. Crop Evol. 71, 3705–3720. doi: 10.1007/s10722-024-01884-w 30311153

[B38] Malyshev L. I. Peshkova G. A. (Eds.) (1987). Flora sibiri. Tom 4: araceae – liliaceae [Flora of siberia. Vol. 4: araceae – liliaceae] (Novosibirsk: Nauka). (In Russian).

[B69] Manafzadeh S. Salvo G. Conti E. (2019). Morphological innovations and vast extensions of mountain habitats triggered rapid diversification within the species-rich Irano-Turanian genus Acantholimon (Plumbaginaceae). Front. Genet. 9, 698. doi: 10.3389/fgene.2018.00698 30745908 PMC6360523

[B70] Mathew B. (1989). The iris (London: Batsford Ltd), 202.

[B71] Matzke N. J. (2014). Model selection in historical biogeography reveals that founder-event speciation is a crucial process in island clades. Syst. Biol. 63, 951–970. doi: 10.1093/sysbio/syu056 25123369

[B72] Matzke N. J. (2022). Statistical comparison of DEC and DEC+ J is identical to comparison of two ClaSSE submodels, and is therefore valid. J. Biogeogr. 49, 1805–1824. doi: 10.31219/osf.io/vqm7r 42226319

[B73] Mavrodiev E. V. Martínez-Azorín M. Dranishnikov P. Crespo M. B. (2014). At least 23 genera instead of one: The case of Iris L. sl (Iridaceae). PloS One 9, e106459. doi: 10.1371/journal.pone.0106459 25170935 PMC4149580

[B74] Meerow A. W. Gideon M. Nakamura K. (2017). Hybridization between ecotypes in a phenotypically and ecologically heterogeneous population of Iris savannarum (Iridaceae) in Florida. Plant Species Biol. 32, 309–322. doi: 10.1111/1442-1984.12158 40046247

[B75] Minh B. Q. Schmidt H. A. Chernomor O. Schrempf D. Woodhams M. D. von Haeseler A. . (2020). IQ-TREE 2: New models and efficient methods for phylogenetic inference in the genomic era. Mol. Biol. Evol. 37, 1530–1534. doi: 10.1093/molbev/msaa015 32011700 PMC7182206

[B77] Moosavi S. J. Mueller M. Gailing O. (2025). Development of new chloroplast microsatellites for Pinus gerardiana and their application in genetic diversity analyses. Ecol. Evol. 15, e71185. doi: 10.1002/ece3.71185 40177694 PMC11961383

[B78] Mykhailenko O. Gudžinskas Z. Kovalyov V. Desenko V. Ivanauskas L. Bezruk I. . (2020). Effect of ecological factors on the accumulation of phenolic compounds in Iris species from Latvia, Lithuania and Ukraine. Phytochem. Anal. 31, 545–563. doi: 10.1002/pca.2918 31965645

[B79] Nazir N. (2013). Immunomodulatory activity of isoflavones isolated from Iris kashmiriana: Effect on T-lymphocyte proliferation and cytokine production in Balb/c mice. Biomedicine Prev. Nutr. 3, 151–157. doi: 10.1016/j.bionut.2012.12.006 38826717

[B80] Nikitina E. V. Ortikov E. Yu B. N. Khalbekova K. U. (2023). Molecular authentication of some rare Iris (Iridaceae) species from Uzbekistan. Plant Sci. Today 10, 444–454. doi: 10.14719/pst.2596

[B81] Omarova B. A. Shults E. E. Zhakipbekov K. S. Abekova А.О. Ishmuratova M. Y. Petrova T. N. . (2024). Biological effects and phytochemical study of the underground part of Iris scariosa Willd. ex Link extract: A new source of bioactive constituents. Fitoterapia 175, 105920. doi: 10.1016/j.fitote.2024.105920 38531480

[B82] Ortikov E. Kurbonalieva M. Alieva K. Khudoyberdieva S. Asatulloev T. Yusupov Z. . (2023). Plastid genomes of four species of Iris from subgenus Scorpiris. Plant Diversity Cent. Asia 2, 102–123. doi: 10.54981/PDCA/vol2_iss2/a4

[B83] Oyuntsetseg D. Nyamgerel N. Baasanmunkh S. Oyuntsetseg B. Urgamal M. Yoon J. W. . (2024). The complete chloroplast genome and phylogentic results support the species position of Swertia banzragczii and Swertia marginata (Gentianaceae) in Mongolia. Bot. Stud. 65, 11. doi: 10.1186/s40529-024-00417-z 38656420 PMC11043322

[B84] Öztaş F. Türkmen A. Öztaş H. Türkmen M. (2024). The medical properties of Iris and its usage in pharmaceutical, perfumery and cosmetic industries. Med. Res. Its Appl. 4, 114–124. doi: 10.9734/bpi/mria/v4/523

[B85] Park I. Yang S. Kim W. J. Song J. H. Lee H. S. Lee H. O. . (2019). Sequencing and comparative analysis of the chloroplast genome of Angelica polymorpha and the development of a novel indel marker for species identification. Molecules 24, 1038. doi: 10.3390/molecules24061038 30875988 PMC6471784

[B86] Parnikoza I. Y. Andreev I. O. Bublyk O. M. Spiridonova K. V. Gołębiewska J. Kubiak M. . (2017). The current state of steppe perennial plants populations: A case study on Iris pumila. Biologia 72, 24–35. doi: 10.1515/biolog-2017-0002 31755547

[B36] Pavlov N. V. (Ed.) (1958). Flora Kazakhstana. Tom 2 [Flora of Kazakhstan. Vol. 2]. Almaty: Izdatel'stvo AN KazSSR. Academy of Sciences of the Kazakh SSR (In Russian).

[B87] Peng C. Guo X. L. Zhou S. D. He X. J. (2023). Backbone phylogeny and adaptive evolution of Pleurospermum sl: New insights from phylogenomic analyses of complete plastome data. Front. Plant Sci. 14, 1148303. doi: 10.3389/fpls.2023.1148303 37063181 PMC10101341

[B88] Peng J. Xie J. Gu Y. Guo H. Zhang S. Huang X. . (2024). Assessing population genetic structure and diversity and their driving factors in Phoebe zhennan populations. BMC Plant Biol. 24, 1091. doi: 10.1186/s12870-024-05810-1 39551749 PMC11572363

[B89] POWO (2026). Plants of the world online. Facilitated by the royal botanic gardens, kew. Published on the internet. Available online at: https://powo.science.kew.org/ (Accessed February 15, 2026).

[B90] Provan J. Powell W. Hollingsworth P. M. (2001). Chloroplast microsatellites: new tools for studies in plant ecology and evolution. Trends Ecol. Evol. 16, 142–147. doi: 10.1016/s0169-5347(00)02097-8 11179578

[B92] Radanova S. S. (2023). Plants in the national symbolism of European countries: A link among countries, cultures, and religions. Asian J. Res. Bot. 6, 158–171.

[B93] Rakhimova N. K. Duschanova G. M. Abdullaeva A. T. Temirov E. E. (2020). Anatomical structure of aboveground and underground organs of the rare endemic species Iris (Juno) magnifica vved. growing under natural conditions of the Zeravshan Ridge, Samarkand Mountains. Am. J. Plant Sci. 11, 1453–1466. doi: 10.4236/ajps.2020.119105

[B95] Ramazanova M. Karzhaubekova Z. Gemejiyeva N. Krasnov K. Shavarda A. Kaidarbekova D. . (2026). Phytochemical analysis, mineral composition and GC-MS profiling of three Iris species from the southeastern part of Kazakhstan. Molecules 31, 643. doi: 10.3390/molecules31040643 41752419 PMC12943009

[B94] Ramazanova M. S. Kurbatova N. V. Gemejiyeva N. G. Aldassugyrova C. Z. (2020). A comparative anatomical and morphological study of vegetative organs of Iris sogdiana Bunge from natural populations of southeastern Kazakhstan. Fundam. Exp. Biol. 99, 109–118.

[B97] Rambaut A. M. (2014). FigTree v1.4.4. Available online at: http://tree.bio.ed.ac.uk/software/figtree/ (Accessed February 20, 2026).

[B96] Rambaut A. Drummond A. J. Xie D. Baele G. Suchard M. A. (2018). Posterior summarization in Bayesian phylogenetics using Tracer 1.7. Syst. Biol. 67, 901–904. doi: 10.1093/sysbio/syy032 29718447 PMC6101584

[B98] Rasul K. S. Majeed H. O. Faraj J. M. Lateef D. D. Tahir N. A. R. (2025). Genetic diversity and relationships among Iris aucheri genotypes determined via ISSR and CDDP markers. Genet. Resour. Crop Evol. 72, 3235–3248. doi: 10.1007/s10722-024-02152-7 30311153

[B99] Raubeson L. A. Peery R. Chumley T. W. Dziubek C. Fourcade H. M. Boore J. L. . (2007). Comparative chloroplast genomics: analyses including new sequences from the angiosperms Nuphar advena and Ranunculus macranthus. BMC Genomics 8, 174. doi: 10.1186/1471-2164-8-174 17573971 PMC1925096

[B100] Raycheva T. Stoyanov K. Denev I. (2011). Genetic diversity and molecular taxonomy study of three genera from Iridaceae family in the Bulgarian flora based on ISSR markers. Biotechnol. Biotechnol. Equip. 25, 2484–2488. doi: 10.5504/BBEQ.2011.0075

[B91] R Core Team (2020). R: A language and environment for statistical computing (Vienna: R Foundation for Statistical Computing). Available online at: https://www.r-project.org/ (Accessed March 04, 2026).

[B102] Ree R. H. Sanmartín I. (2018). Conceptual and statistical problems with the DEC+J model of founder-event speciation and its comparison with DEC via model selection. J. Biogeogr. 45, 741–749. doi: 10.1111/jbi.13173 40046247

[B103] Rodionenko G. I. (1987). The genus Iris L. (questions of morphology, biology, evolution and systematics) (London: The British Iris Society), 222. English translation.

[B104] Roguz K. Gallagher M. K. Senden E. Bar-Lev Y. Lebel M. Heliczer R. . (2020). All the colors of the rainbow: diversification of flower color and intraspecific color variation in the genus Iris. Front. Plant Sci. 11, 569811. doi: 10.3389/fpls.2020.569811 33154761 PMC7588356

[B105] Ronquist F. Teslenko M. Van Der Mark P. Ayres D. L. Darling A. Höhna S. . (2012). MrBayes 3.2: Efficient Bayesian phylogenetic inference and model choice across a large model space. Syst. Biol. 61, 539–542. doi: 10.1093/sysbio/sys029 22357727 PMC3329765

[B106] Salimbahrami M. Saeidi H. Bagheri A. Wilson C. A. Schneeweiss G. M. (2026). Biogeography and diversification patterns in the Irano-Turanian biodiversity hotspots inferred from a molecular phylogeny of the subendemic Iris subgenus Scorpiris (Iridaceae). Nord. J. Bot., e05014. doi: 10.1002/njb.05014 41531421

[B107] Sauquet H. (2013). A practical guide to molecular dating. C.R. Palevol 12, 355–367. doi: 10.1016/j.crpv.2013.07.003 38826717

[B108] Sennikov A. Khassanov F. Ortikov E. Kurbonaliyeva M. Tojibaev K. S. (2023). The genus Iris L. sl (Iridaceae) in the Mountains of Central Asia biodiversity hotspot. Plant Diversity Cent. Asia 2, 1–104. doi: 10.54981/pdca/vol3_iss1/a

[B109] Shen L. Chen S. Liang M. Qu S. Feng S. Wang D. . (2024). Comparative analysis of codon usage bias in chloroplast genomes of ten medicinal species of Rutaceae. BMC Plant Biol. 24, 424. doi: 10.1186/s12870-024-04999-5 38764045 PMC11103831

[B110] Shin J. S. Hong S. W. Lee J. G. Lee Y. M. Kim D. W. Kim J. E. . (2011). An ethanol extract of Iris nertschinskia induces p53-dependent apoptosis in the MCF7 human breast cancer cell line. Int. J. Mol. Med. 27, 401–405. doi: 10.1093/rheumatologykeg209 21240456

[B111] Shomurodov H. F. Saribaeva S. U. Abduraimov O. S. Khayitov R. S. Sayfullaev A. F. (2021). The current state of iris Hippolyti's (Vved.) Kamelin population in Uzbekistan. Ann. Rom. Soc Cell Biol. 25, 6589–6597.

[B112] Soares A. E. Schrago C. G. (2012). The influence of taxon sampling and tree shape on molecular dating: an empirical example from Mammalian mitochondrial genomes. Bioinf. Biol. Insights 6, BBI-S9677. doi: 10.4137/bbi.s9677 22693422 PMC3370833

[B113] Song B. N. Liu C. K. Zhao A. Q. Tian R. M. Xie D. F. Xiao Y. L. . (2024a). Phylogeny and diversification of genus Sanicula L. (Apiaceae): novel insights from plastid phylogenomic analyses. BMC Plant Biol. 24, 70. doi: 10.1186/s12870-024-04750-0 38263006 PMC10807117

[B114] Song Y. Wang L. Zhang L. Li J. Teng Y. Zhang Z. . (2024b). Unified assembly of Chloroplast genomes: A comparative study of grapes representing global geographic diversity. Horticulturae 10, 1218. doi: 10.3390/horticulturae10111218 30654563

[B115] Sun M. Z. Li M. R. Shi F. X. Li L. Liu Y. Li L. F. . (2012). Genomic and EST‐derived microsatellite markers for Iris laevigata (Iridaceae) and other congeneric species. Am. J. Bot. 99, e286–e288. doi: 10.3732/ajb.1100608 22739712

[B116] Tao K. Tao L. Huang J. Duan H. Luo Y. Li L. (2024). Complete chloroplast genome structural characterization of two Aerides (Orchidaceae) species with a focus on phylogenetic position of Aerides flabellata. BMC Genomics 25, 552. doi: 10.1186/s12864-024-10458-0 38825700 PMC11145882

[B117] Terzoli S. Abbruzzese G. Beritognolo I. Sabatti M. Valentini R. Kuzminsky E. (2014). Genetic characterization of a Tamarix spp. germplasm collection in Italy. Botany 92, 360–369. doi: 10.1139/cjb-2013-0270 34819996

[B118] Tillich M. Lehwark P. Pellizzer T. Ulbricht-Jones E. S. Fischer A. Bock R. . (2017). GeSeq – versatile and accurate annotation of organelle genomes. Nucleic Acids Res. 45, W6–W11. doi: 10.1093/nar/gkx391 28486635 PMC5570176

[B119] Tojibaev K. S. Yusupov Z. Sennikov A. N. Ibragimov A. Ortikov E. Asatulloev T. (2025). Iris anvarbekii (Iridaceae), a new species of I. subg. Scorpiris from the southern Pamir-Alay in Uzbekistan. Nord. J. Bot. 2025, e04860. doi: 10.1002/njb.04860 41531421

[B120] Turgunov M. D. Pechenitsyn V. P. Beshko N. Y. Uralov A. I. Abdullaev D. A. (2019). Biological features of rare species of Iridaceae Juss. family in flora of Uzbekistan ex situ. Acta Biologica Sibirica 5, 17–22. doi: 10.4258/abs.v5.i2.5926 41868777

[B121] Vereecken N. J. Wilson C. A. Hötling S. Schulz S. Banketov S. A. Mardulyn P. (2012). Pre-adaptations and the evolution of pollination by sexual deception: Cope's rule of specialization revisited. Proc. R. Soc. B. Biol. Sci. 279, 4786–4794. doi: 10.1098/rspb.2012.1804 23055065 PMC3497092

[B122] Verma V. Kumar A. Priti Seema Thakur M. Bhargava B. (2022). Meta-Topolin mediated *in vitro* propagation in an ornamentally important crop Iris× hollandica Tub. cv. professor Blaauw and genetic fidelity studies using SCoT markers. Plant Cell. Tissue Organ. Culture (PCTOC) 151, 681–694. doi: 10.1007/s11240-022-02383-5 30311153

[B124] Volis S. Depalle F. Khassanov F. Yusupov Z. Deng T. (2024). Oncocyclus irises: Phylogeny, evolutionary history and revised taxonomy based on complete chloroplast genome sequences. Plant Diversity Cent. Asia 3, 1–66.

[B123] Volis S. Zhang Y. Deng T. Yusupov Z. (2022). Dark-colored Oncocyclus irises in Israel analyzed by AFLP, whole chloroplast genome sequencing and species distribution modeling. Israel J. Ecol. Evol. 68, 43–53. doi: 10.1163/22244662-bja10037

[B126] Wang Z. Cai Q. Wang Y. Li M. Wang C. Wang Z. . (2022). Comparative analysis of codon bias in the chloroplast genomes of Theaceae species. Front. Genet. 13, 824610. doi: 10.3389/fgene.2022.824610 35360853 PMC8961065

[B125] Wang X. Kraatz B. Meng J. Carrapa B. DeCelles P. Clementz M. . (2016). Central Asian aridification during the late Eocene to early Miocene inferred from preliminary study of shallow marine–eolian sedimentary rocks from northeastern Tajik Basin. Sci. China Earth Sci. 59, 1242–1257. doi: 10.1007/s11430-016-5282-z 30311153

[B127] Weber T. Jakše J. Sladonja B. Hruševar D. Landeka N. Brana S. . (2020). Molecular study of selected taxonomically critical taxa of the genus Iris L. from the broader Alpine-Dinaric area. Plants 9, 1229. doi: 10.3390/plants9091229 32961899 PMC7570032

[B128] Wicke S. Schneeweiss G. M. Depamphilis C. W. Müller K. F. Quandt D. (2011). The evolution of the plastid chromosome in land plants: gene content, gene order, gene function. Plant Mol. Biol. 76, 273–297. doi: 10.1007/s11103-011-9762-4 21424877 PMC3104136

[B129] Wilson C. A. (2009). Phylogenetic relationships among the recognized series in Iris section Limniris. Syst. Bot. 34, 277–284. doi: 10.1600/036364409788606316 40631773

[B130] Wilson C. A. (2011). Subgeneric classification in Iris re-examined using chloroplast sequence data. Taxon. 60, 27–35. doi: 10.1002/tax.601004 41531421

[B132] Wilson C. A. (2017). Sectional relationships in the Eurasian bearded Iris (subgen. Iris) based on phylogenetic analyses of sequence data. Syst. Bot. 42, 392–401. doi: 10.1600/036364417X695970 40631773

[B131] Wilson C. A. Padiernos J. Sapir Y. (2016). The royal irises (Iris subg. Iris sect. Oncocyclus): Plastid and low‐copy nuclea data contribute to an understanding of their phylogenetic relationships. Taxon. 65, 35–46. doi: 10.12705/651.3 42091727

[B133] Wollenweber E. Stevens J. F. Klimo K. Knauft J. Frank N. Gerhäuser C. (2003). Cancer chemopreventive *in vitro* activities of isoflavones isolated from Iris germanica. Planta Med. 69, 15–20. doi: 10.1055/s-2003-37030 12567273

[B134] Wu C. Liu R. Li C. Shang M. Zhang Y. Bai K. . (2025). Comparative analysis of codon usage bias and phylogenetic relationships in chloroplast genomes across 49 Dendrobium species. BMC Plant Biol. 25, 1411. doi: 10.1186/s12870-025-07395-9 41120874 PMC12538735

[B135] Xiao X. Chen J. Ran Z. Huang L. Li Z. (2025). Comparative analysis of complete Chloroplast genomes and phylogenetic relationships of 21 sect. Camellia (Camellia L.) plants. Genes 16, 49. doi: 10.3390/genes16010049 39858596 PMC11764880

[B136] Xu S. Teng K. Zhang H. Gao K. Wu J. Duan L. . (2023). Chloroplast genomes of four Carex species: Long repetitive sequences trigger dramatic changes in chloroplast genome structure. Front. Plant Sci. 14. doi: 10.3389/fpls.2023.1100876 36778700 PMC9911286

[B137] Yang Z. (2007). PAML 4: phylogenetic analysis by maximum likelihood. Mol. Biol. Evol. 24, 1586–1591. doi: 10.1093/molbev/msm088 17483113

[B138] Ye L. Shavvon R. S. Qi H. Wu H. Fan P. Shalizi M. N. . (2024). Population genetic insights into the conservation of common walnut (Juglans regia) in Central Asia. Plant Divers. 46, 600–610. doi: 10.1016/j.pld.2024.06.001 39290885 PMC11403145

[B139] Yermagambetova M. Almerekova S. Turginov O. Sultangaziev O. Abugalieva S. Turuspekov Y. (2023). Genetic diversity and population structure of Juniperus seravschanica Kom. collected in Central Asia. Plants 12, 2961. doi: 10.3390/plants12162961 37631172 PMC10459705

[B140] Yermagambetova M. Imanbayeva A. Ishmuratova M. Sumbembayev A. Almerekova S. (2025). Characterization of the four rosa L. Species from Kazakhstan based on complete plastomes and nuclear ribosomal internal transcribed spacer (ITS) sequences. Genes 16, 852. doi: 10.3390/plants13101332 40869900 PMC12386023

[B141] Yu Y. Blair C. He X. (2020). RASP 4: ancestral state reconstruction tool for multiple genes and characters. Mol. Biol. Evol. 37, 604–606. doi: 10.1093/molbev/msz257 31670774

[B143] Zhang G. Han Y. Wang H. Wang Z. Xiao H. Sun M. (2021). Phylogeography of Iris loczyi (Iridaceae) in Qinghai–Tibet Plateau revealed by chloroplast DNA and microsatellite markers. AoB Plants 13, plab070. doi: 10.1007/978-981-32-9034-1_4 34876969 PMC8643446

[B144] Zhang Y. Tian L. Lu C. (2023). Chloroplast gene expression: Recent advances and perspectives. Plant Commun. 4, 100611. doi: 10.1016/j.xplc.2023.100611 37147800 PMC10504595

[B142] Zhang M. L. Zeng X. Q. Sanderson S. C. Byalt V. V. Sukhorukov A. P. (2017). Insight into Central Asian flora from the Cenozoic Tianshan montane origin and radiation of Lagochilus (Lamiaceae). PloS One 12, e0178389. doi: 10.1371/journal.pone.0178389 28931016 PMC5606930

[B145] Zhu M. Feng P. Ping J. Li J. Su Y. Wang T. (2021). Phylogenetic significance of the characteristics of simple sequence repeats at the genus level based on the complete chloroplast genome sequences of Cyatheaceae. Ecol. Evol. 11, 14327–14340. doi: 10.22541/au.161587690.08363674/v1 34707858 PMC8525152

