# Supplementary material for: Phylogenomics, divergence time estimation, and biogeography of Iris species from Kazakhstan using plastome sequence analysis
Source: Front Plant Sci. 2026 Jun 17;17:1860819. doi: 10.3389/fpls.2026.1860819 (PMC13318877; doi:10.3389/fpls.2026.1860819)
Supplement: Supplementary file 1 [file Table1.docx]

**Supplementary Table S1.** Key diagnostic morphological features of 14 *Iris* species

| № | Species | Morphological description |
| --- | --- | --- |
| 1 | *I. glaucescens* | Rhizome thick, up to 1.5 cm in diameter. Leaves all basal, sword-shaped, glaucous; stem not exceeding leaf height. Bracts three, leathery, scarious. Flowers two, on short pedicels, lilac-purple. Perianth segments obovate, bearing a band of white hairs; inner segments equal in length to outer ones but narrower. Perianth tube completely concealed by bracts, 1.5-3 times longer than ovary. Capsule fusiform. Seeds dark brown, rugose |
| 2 | *I. halophila* | Basal leaves broad, sword-shaped, rigid. Stem slightly compressed. Bracts two, herbaceous, acute, apically scarious. Flowers two to four, yellow, sometimes pale bluish. Outer perianth segments horizontal, reflexed, elliptic, constricted; inner segments erect, lanceolate. Perianth tube subequal in length to ovary. Capsule beaked. Seeds brown, compressed, sometimes angular |
| 3 | *I. lactea* | Shoot bases with rigid, dark brown, thread-like shredded remains of sheaths. Stems numerous, 10-20 cm tall. Basal leaves 15-20 cm long, 3-5 mm wide, glaucous-green. Involucral bracts broadly scarious, becoming papery in fruit, usually not reaching the capsule base. Flowers one or two, pale blue, rarely white. Outer perianth segments widest at mid-limb, erect to suberect, scarcely differing in width from inner segments. Capsule 3-6 cm long, fusiform or oblong, with a beak 3-7 mm long |
| 4 | *I. pumila* | Rhizome branched, up to 10 mm thick, producing clusters of shoots. Stem almost undeveloped. Plants 10-15 cm tall. All leaves basal, slightly glaucous, long-lanceolate, 8-16 cm long, 6-11 mm wide. Bracts narrow, apically scarious. Flowers solitary, blue, violet, or yellow. Outer perianth segments oblong, cuneately narrowed toward base, with a dense beard of multicellular hairs; inner perianth segments almost equal in length to outer but wider and emarginate. Perianth tube 6–8 cm long, twice as long as the perianth segments. Stigmas entire, expanded, almost sessile. Capsule trigonous, apically acute. Seeds ca. 5 mm long, wrinkled, dark brown, irregularly ovoid |
| 5 | *I. ruthenica* | Rhizome cord-like, creeping, branched, 2-3 mm thick, covered with brown fibrous leaf remains. Stem thin, 6-18 cm tall, with 1-3 small stem-clasping leaves in lower half. Basal leaves longer than stem, linear, long-acuminate, 15-50 cm long, 3-6 mm wide. Bracts two, elliptic-lanceolate, 3-4 cm long. Flower solitary, fragrant, blue-violet. Perianth tube 10-15 mm long, 2.5 times shorter than limb. Outer perianth segments oblong, narrowed at base, rounded and usually notched at apex; inner segments slightly shorter and 2-3 times narrower, obversely linear-cuneate. Capsule hemispherical, beakless. Seeds about 2.5 mm long, almost trigonous, with an appendage |
| 6 | *I. sibirica* | Rhizome thin, covered with fibrous remains of previous year's leaves. Plants 35-80 cm tall. Stem erect, cylindrical, hollow, nearly leafless in upper part. Leaves narrow-linear, sword-shaped, 3-8 mm wide, shorter than flowering stem. Bracts brown, apically scarious. Flowers solitary, sessile, terminal, or 2-3 in axils, sometimes in a racemose inflorescence, fragrant, blue-violet, rarely white. Outer perianth segments obovate, blue on claw, with brown or violet venation; inner perianth segments erect, elliptic-ovate, darker and larger than style branches |
| 7 | *I. sogdiana* | 25-50 cm tall, 1-1.5 cm thick, covered in upper part with rigid brown leaf remains. Stem slightly compressed, with 3-5 lanceolate leaves not exceeding stem length. Basal leaves slightly longer than stem, 8-16 mm wide, long-lanceolate, acute, grayish-green. Bracts broadly lanceolate, acute, with white-scarious margins. Flowers 2-4, pale yellow or whitish, on pedicels shorter than or subequal to perianth. Outer perianth segments reflexed, almost elliptic, with upper part rounded-ovate then constricted into a narrow oblong portion longer than the upper part; inner perianth segments erect, slightly shorter and narrower than outer, oblong-elliptic, obtuse. Capsule 4-5.5 cm long, oval or oblong-oval. Seeds ca. 6 mm, brownish, shiny coat |
| 8 | *I. songarica* | Rhizome thin, dark; old leaf remains forming a spiral fiber cluster. Plants 40-80 cm tall. Stem solid, up to 40 cm tall. Leaves up to 10 cm long, 4-6 mm wide, with prominent veins. Bracts leathery, pale green. Peduncles 1-4, each bearing 2-3 flowers, pedicel ca. 1 cm. Perianth tube 4-6 cm long. Outer perianth segments with elliptic blade constricted from claw; claw pale blue with reddish-purple spots, blade bluish. Inner segments obovate-lanceolate, cuneate, with red-purple spots. Style branches narrow, overlapping; stigmas bilobed with two triangular teeth.. Capsule oblong. Seeds cylindrical, dark brown, wrinkled |
| 9 | *I. tenuifolia* | Rhizome rather thin, many-headed at apex, bearing brownish-straw, hard, long (5-11 cm) shredded remains of leaf sheaths densely covering leaf tufts. Stem short, 2-4 cm tall. Plant is 20-40 cm tall. Basal leaves 20-40 cm long, 1-1.5 mm wide, narrow, linear, grayish-green, hard. Bracts 3-4, lanceolate, folded lengthwise, one obtuse, others acute. Flowers 2-3, light blue or blue-violet. Perianth tube thin-filiform, 1.5-2 times longer than limb segments. Outer perianth segments glabrous, oblong, obtuse, gradually narrowed into a long wide claw; inner segments slightly shorter and narrower than outer. Style lobes oblong, apically narrowed. Capsule broadly oval, 3-angled, 2.5-3 cm long, with a short beak. Seeds irregular, oblong, 4-6 mm long, dark brown, wrinkled |
| 10 | *I. kolpakowskiana* | Bulb covered with fibrous remains of leaf sheaths forming a net. Plants 10-25 cm tall. Stem not developed; scape one-flowered. Basal leaves 3-6, enclosed at base in a single scarious sheath 3-6 cm long, linear, 1-1.5 mm wide, apically recurved and acute. Bracts lanceolate, acute, green. Perianth tube 3-6 cm long. Outer perianth segments lanceolate-oblong, narrowed toward base, externally bluish- or blue-violet, with a white spot at base and a bright yellow crest; claw yellow. Inner perianth segments oblong, uniformly pale purple. Style branches pale purple; lobes narrow, acute. Stigma entire |
| 11 | *I. kuschakewiczii* | Rhizome thickened, cord-like; bulb well-developed, ca. 1.5 cm thick. Stem short, ca. 3 cm. Leaves 4-5, dark green, broadly margined, scabrous; lower leaves 1-1.5 cm wide. Flowers 1-3(4), pale violet, odorless. Perianth tube 3.5-4.5 cm long. Outer perianth segments 3.5-4 cm long; claw with 5 dark violet interrupted veins; blade 7 × 12 mm, white with dark violet spot; crest white, dentate. Inner perianth segments 1-1.5 cm long, with three-lobed acute blade. Style branches obliquely triangular, 4 × 10 mm; stigmas semicircular. |
| 12 | *I. orchioides* | Plant 10-20 cm tall. Bulb ca. 2 cm thick. Leaves light green, falcate, margined; lower leaves 2-3, up to 5 cm wide. Flowers 3-4(8), pale yellow, becoming violet after anthesis. Perianth tube 3-6 cm long, often violet. Outer perianth segments 4.5 cm long; claw strongly winged; blade 15-18 × 10-12 mm, with a large dark yellow spot; crest dark yellow, entire and dentate anteriorly, dissected posteriorly. Inner perianth segments 10-15 mm long, with three-lobed acute blade. Style branches obliquely triangular, 9-10 × 5-6 mm; stigmas reniform-obverse |
| 13 | *I. subdecolorata* | Plant 5-10 cm tall. Bulb ca. 1.5 cm thick. Leaves 4-6, dark green, glossy, falcate, broadly margined; lower leaves 8-20 mm wide. Flowers 1-3, translucent, almost colorless, odorless. Perianth tube ca. 4.5 cm long. Outer perianth segments 4-4.5 cm long; claw with 2 dirty violet veins; blade 14-16 × 7-10 mm, with dirty green spots. Inner perianth segments 15-20 mm long, with three-lobed blade. Style branches 11-13 × 4 mm; stigmas semicircular. Anthers whitish; filaments bluish |
| 14 | *I. willmottiana* | Bulb stout, globose (Juno type). Stem 15-20 cm tall, bearing 4-6(9) sessile flowers in leaf axils. Leaves ca. 8, deep green, glossy, falcate, with white horny margin. Spathe valves narrow, 2.5-5 cm long. Perianth tube ca. 5 cm long, triangular. Outer perianth segments: haft pale reddish-purple with blue-violet veins; blade blue with white patch marked by darker blotches. Inner perianth segments small, depressed, with pointed tooth and two rounded projections. Style keeled; crests small, erect; stigma entire, oblong. Filaments whitish. Anthers and pollen cream. Capsule trigonal, oblong, with papery walls. Seeds spherical, brown, wrinkled |
