# Supplementary material for: Phylogenomics, divergence time estimation, and biogeography of Iris species from Kazakhstan using plastome sequence analysis
Source: Front Plant Sci. 2026 Jun 17;17:1860819. doi: 10.3389/fpls.2026.1860819 (PMC13318877; doi:10.3389/fpls.2026.1860819)
Supplement: Supplementary file 2 [file Table2.docx]

**Supplementary Table S2.** GPS coordinates and raw data accession information for 14 *Iris* species from Kazakhstan

| № | Species | Coordinates | Collection year | Voucher number | GenBank Accession Number | BioProject | BioSample | SRA |
| --- | --- | --- | --- | --- | --- | --- | --- | --- |
| 1 | *I. glaucescens* | 48.62351095 N  66.99011962 E | 2024 | IPBB40030301 | PX505513 | PRJNA1471469 | SAMN60452615 | SRR38877287 |
| 2 | *I. halophila* | 48.60709567 N  66.97906615 E | 2024 | IPBB40030701 | PX505514 | PRJNA1471469 | SAMN60452616 | SRR38877286 |
| 3 | *I. lactea* | 50.76111708 N  75.70451059 E | 2024 | IPBB40031601 | PX505515 | PRJNA1471469 | SAMN60452617 | SRR38877282 |
| 4 | *I. pumila* | 51.265583 N  53.128167 E | 2025 | IPBB40032101 | PX505517 | PRJNA1471469 | SAMN60452618 | SRR38877281 |
| 5 | *I. sibirica* | 53.34123309 N  75.47055843 E | 2024 | IPBB40031501 | PX505518 | PRJNA1471469 | SAMN60452619 | SRR38877280 |
| 6 | *I. sogdiana* | 70.542861 N  42.413639 E | 2017 | IPBB40030502 | PX505519 | PRJNA1471469 | SAMN60452620 | SRR38877279 |
| 7 | *I. songarica* | 43.931388 N  75.455048 E | 2025 | IPBB40031802 | PX505520 | PRJNA1471469 | SAMN60452621 | SRR38877278 |
| 8 | *I. tenuifolia* | 46.026667 N  73.199722 E | 2024 | IPBB40030603 | PX505521 | PRJNA1471469 | SAMN60452622 | SRR38877277 |
| 9 | *I. ruthenica* | 42.989115 N  78.324688 E | 2025 | IPBB40032301 | PX915248 | PRJNA1471469 | SAMN60452625 | SRR38877285 |
| 10 | *I. willmottiana* | 42.48264 N  70.039159 E | 2025 | IPBB40031901 | PX505522 | PRJNA1471469 | SAMN60452623 | SRR38877276 |
| 11 | *I. orchioides* | 42.317443 N  69.247757 E | 2025 | IPBB40031202 | PX505524 | PRJNA1471469 | SAMN60452624 | SRR38877275 |
| 12 | *I. kuschakewiczii* | 43.845957 N  76.343987 E | 2025 | IPBB40031105 | PX505525 | PRJNA1338957 | SAMN52588717 | SRR35767050 |
| 13 | *I. subdecolorata* | 43.313375 N  70.831328 E | 2025 | IPBB40032006 | PX915249 | PRJNA1471469 | SAMN60452627 | SRR38877283 |
| 14 | *I.kolpakowskiana* | 43.272982 N  75.781057 E | 2025 | IPBB40031705 | PX910022 | PRJNA1471469 | SAMN60452626 | SRR38877284 |
