# Supplementary material for: Phylogenomics, divergence time estimation, and biogeography of Iris species from Kazakhstan using plastome sequence analysis
Source: Front Plant Sci. 2026 Jun 17;17:1860819. doi: 10.3389/fpls.2026.1860819 (PMC13318877; doi:10.3389/fpls.2026.1860819)
Supplement: Supplementary file 3 [file Table3.docx]

**Supplementary Table S3.** The list of genes annotated in 14 *Iris* plastomes

| **Category** | **Gene type** | **Gene** |
| --- | --- | --- |
| Self-replication | Transfer RNA | *trnA-UGC* (x2)**, trnC-GCA, trnD-GUC, trnE-UUC, trnF-GAA, trnfM-CAU, trnG-GCC, trnG-UCC*, trnH-GUG* (x2), *trnI-CAU, trnI-GAU** (x2), *trnK-UUU*, trnL-CAA* (x2), *trnL-CAU, trnL-UAA*, trnL-UAG, trnM-CAU*, *trnN-GUU* (x2), *trnP-UGG, trnQ-UUG, trnR-ACG* (x2), *trnR-UCU, trnS-GCU, trnS-GGA, trnS-UGA, trnT-GGU, trnT-UGU, trnV-GAC* (x2), *trnV-UAC*, trnW-CCA, trnY-GUA* |
|  | Ribosomal RNA | *rrn16* (x2)*, rrn23* (x2)*, rrn4.5* (x2)*, rrn5* (x2) |
|  | Small ribosomal subunit | *rps11, rps12*** (x2)*, rps14, rps15, rps16*, rps18, rps19* (x2)*, rps2, rps3, rps4, rps7* (x2)*, rps8* |
|  | Large ribosomal subunit | *rpl14, rpl16*, rpl2** (x2)*, rpl20, rpl22, rpl23* (x2)*, rpl32, rpl33, rpl36* |
|  | RNA polymerase subunit | *rpoA, rpoB, rpoC1*, rpoC2* |
|  | Translational initiation factor | *infA* |
| Photosynthesis genes | Large subunit of rubisco | *rbcL* |
|  | Photosystem I | *psaA, psaB, psaC, psaI, psaJ* |
|  | Photosystem II | *psbA, psbB, psbC, psbD, psbE, psbF, psbH, psbI, psbJ, psbK, psbL, psbM, psbN, psbT, psbZ* |
|  | ATP synthase | *atpA, atpB, atpE, atpF*, atpH, atpI* |
|  | Subunits of cytochrome | *petA, petB*, petD*, petG, petL, petN* |
|  | NADH dehydrogenase | *ndhA*, ndhB** (x2)*, ndhC, ndhD, ndhE, ndhF, ndhG, ndhH, ndhI, ndhJ, ndhK* |
| Others | Maturase | *matK* |
|  | Protease | *clpP*** |
|  | C-type cytochrome synthesis | *ccsA* |
|  | envelope membrane protein | *cemA* |
|  | acetyl-CoA | *accD* |
| Unknown genes | Hypothetical gene reading frame | *ycf1* (x2)*, ycf2* (x2)*, ycf3**, ycf4* |

*Note:* One or two asterisks indicate one or two intron-containing genes, respectively, (x2) indicates duplicated genes.
