# Supplementary material for: Phylogenomics, divergence time estimation, and biogeography of Iris species from Kazakhstan using plastome sequence analysis
Source: Front Plant Sci. 2026 Jun 17;17:1860819. doi: 10.3389/fpls.2026.1860819 (PMC13318877; doi:10.3389/fpls.2026.1860819)
Supplement: Supplementary file 4 [file Table4.docx]

**Supplementary Table S4.** The list of highly variable regions among studied 14 *Iris* plastomes

| **Variable Region** | **Length** | **Variable (Polymorphic) Sites** | **Parsimony Informative Sites** | **Nucleotide Diversity** | **Region** |
| --- | --- | --- | --- | --- | --- |
| *rps16* | 765 | 102 | 39 | 0,05346 | genic |
| *rps16-trnQ(UUG)* | 688 | 96 | 42 | 0,05031 | intergenic |
| *trnS(GCU)-trnG(UCC)* | 695 | 95 | 46 | 0,04979 | intergenic |
| *trnG(UCC)* | 714 | 93 | 44 | 0,04874 | genic |
| *trnY(GUA)* | 644 | 89 | 49 | 0,04664 | genic |
| *trnD(GUC)* | 643 | 92 | 50 | 0,04822 | genic |
| *rpl32* | 2010 | 106 | 48 | 0,05555 | genic |
| *rps15* | 920 | 114 | 52 | 0,05975 | genic |
| *clpP* | 683 | 120 | 49 | 0,06289 | genic |
| *ycf1* | 895 | 155 | 65 | 0,08123 | genic |
