# Supplementary material for: Phylogenomics, divergence time estimation, and biogeography of Iris species from Kazakhstan using plastome sequence analysis
Source: Front Plant Sci. 2026 Jun 17;17:1860819. doi: 10.3389/fpls.2026.1860819 (PMC13318877; doi:10.3389/fpls.2026.1860819)
Supplement: Supplementary file 5 [file Table5.docx]

**Supplementary Table S5.** The list of SSRs identified in 14 *Iris* plastomes

| Repeats | *glaucescens* | *halophila* | *lactea* | *pumila* | *sibirica* | *sogdiana* | *songarica* | *tenuifolia* | *ruthenica* | *willmottiana* | *orchioides* | *kuschakewiczii* | *subdecolorata* | *kolpakowskiana* | **Total** |
| --- | --- | --- | --- | --- | --- | --- | --- | --- | --- | --- | --- | --- | --- | --- | --- |
| A/T | 134 | 106 | 95 | 131 | 106 | 106 | 88 | 87 | 81 | 125 | 124 | 125 | 125 | 92 | **1525** |
| C/G | 1 | 6 | 4 | 1 | 6 | 6 | 9 | 6 | 4 | 1 | 2 | 1 | 2 | 3 | **52** |
| AC/GT | 2 | 1 | 2 | 2 | 1 | 1 | 1 | 1 | 3 | 2 | 2 | 2 | 2 | 4 | **26** |
| AG/CT | 16 | 12 | 12 | 16 | 12 | 12 | 13 | 13 | 14 | 15 | 16 | 15 | 15 | 16 | **197** |
| AT/AT | 34 | 30 | 30 | 34 | 30 | 30 | 31 | 31 | 28 | 34 | 33 | 39 | 35 | 26 | **445** |
| AAG/CTT | 2 | 1 | 2 | 2 | 1 | 1 | 1 | 2 | 2 | 3 | 3 | 3 | 3 | 2 | **28** |
| AAT/ATT | 1 | 2 | 1 | 1 | 2 | 2 | 1 | 1 | 1 | 1 | 1 | 1 | 1 | 1 | **17** |
| AAAT/ATTT | 3 | 5 | 3 | 3 | 5 | 5 | 5 | 4 | 5 | 4 | 4 | 4 | 4 | 4 | **58** |
| AATG/ATTC | 1 | 1 | 1 | 1 | 1 | 1 | 1 | 1 | 1 | 1 | 1 | 1 | 1 | 1 | **14** |
| AAAC/GTTT |  | 1 | 1 |  | 1 | 1 | 1 |  | 1 |  |  |  |  | 1 | **7** |
| AATC/ATTG |  |  |  |  |  |  | 8 |  |  |  |  |  |  |  | **8** |
| AATT/AATT |  |  |  |  |  |  |  |  | 1 |  |  |  |  |  | **1** |
| ACAT/ATGT |  |  |  |  |  |  | 1 |  |  |  |  |  |  |  | **1** |
| AACTT/AAGTT | 1 |  | 1 | 1 |  |  |  | 1 | 1 | 1 | 1 | 1 | 1 |  | **9** |
| AAAAT/ATTTT |  |  | 1 |  |  |  |  |  |  |  |  |  |  |  | **1** |
| AAAGG/CCTTT |  |  | 1 |  |  |  |  |  |  |  |  |  |  | 2 | **3** |
| AAGAC/CTTGT |  |  | 1 |  |  |  |  |  |  |  |  |  |  |  | **1** |
| AACAAG/CTTGTT |  |  |  |  |  |  |  |  |  | 1 | 1 | 1 | 1 |  | **4** |
| AAAAAG/CTTTTT |  |  |  |  |  |  |  | 2 |  |  |  |  |  |  | **2** |
| AATATC/ATATTG |  |  |  |  |  |  | 1 |  |  |  |  |  |  |  | **1** |
| AATCAT/ATGATT |  |  |  |  |  |  | 1 |  |  |  |  |  |  |  | **1** |
| **Total** | **195** | **165** | **155** | **192** | **165** | **165** | **162** | **149** | **142** | **188** | **188** | **193** | **190** | **152** | **2401** |
