# Supplementary material for: Phylogenomics, divergence time estimation, and biogeography of Iris species from Kazakhstan using plastome sequence analysis
Source: Front Plant Sci. 2026 Jun 17;17:1860819. doi: 10.3389/fpls.2026.1860819 (PMC13318877; doi:10.3389/fpls.2026.1860819)
Supplement: Supplementary file 6 [file Table6.docx]

**Supplementary Table S6.** RSCU values of 14 *Iris* plastome protein coding genes

| Amino  Acid | Sym  bol | Codon | glauce  scens | halo  phila | kolpakow  skiana | kuscha  kewiczii | lac  tea | orchi  oides | pum  ila | ruthe  nica | sibi  rica | sogdi  ana | songa  rica | subde  colorata | tenui  folia | willmo  ttiana |
| --- | --- | --- | --- | --- | --- | --- | --- | --- | --- | --- | --- | --- | --- | --- | --- | --- |
| * | Ter | UAA | 1,31 | 1,21 | 1,21 | 1,31 | 1,24 | 1,31 | 1,31 | 1,21 | 1,24 | 1,21 | 1,24 | 1,31 | 1,28 | 1,31 |
| * | Ter | UAG | 0,93 | 0,97 | 0,93 | 0,97 | 0,97 | 0,93 | 0,93 | 1 | 0,97 | 0,97 | 0,93 | 0,97 | 0,93 | 0,97 |
| * | Ter | UGA | 0,76 | 0,83 | 0,86 | 0,72 | 0,79 | 0,76 | 0,76 | 0,79 | 0,79 | 0,83 | 0,83 | 0,72 | 0,79 | 0,72 |
| A | Ala | GCA | 1,15 | 1,12 | 1,13 | 1,13 | 1,14 | 1,13 | 1,16 | 1,12 | 1,11 | 1,12 | 1,14 | 1,12 | 1,1 | 1,12 |
| A | Ala | GCC | 0,64 | 0,64 | 0,62 | 0,66 | 0,7 | 0,65 | 0,65 | 0,67 | 0,65 | 0,64 | 0,65 | 0,66 | 0,67 | 0,66 |
| A | Ala | GCG | 0,41 | 0,44 | 0,44 | 0,44 | 0,42 | 0,44 | 0,41 | 0,44 | 0,44 | 0,44 | 0,46 | 0,45 | 0,47 | 0,45 |
| A | Ala | GCU | 1,79 | 1,8 | 1,81 | 1,77 | 1,74 | 1,77 | 1,78 | 1,77 | 1,8 | 1,8 | 1,75 | 1,77 | 1,76 | 1,77 |
| C | Cys | UGC | 0,42 | 0,44 | 0,44 | 0,43 | 0,42 | 0,43 | 0,41 | 0,46 | 0,43 | 0,44 | 0,43 | 0,43 | 0,43 | 0,42 |
| C | Cys | UGU | 1,58 | 1,56 | 1,56 | 1,57 | 1,58 | 1,57 | 1,59 | 1,54 | 1,57 | 1,56 | 1,57 | 1,57 | 1,57 | 1,58 |
| D | Asp | GAC | 0,39 | 0,4 | 0,4 | 0,38 | 0,4 | 0,38 | 0,39 | 0,4 | 0,4 | 0,4 | 0,37 | 0,38 | 0,38 | 0,38 |
| D | Asp | GAU | 1,61 | 1,6 | 1,6 | 1,62 | 1,6 | 1,62 | 1,61 | 1,6 | 1,6 | 1,6 | 1,63 | 1,62 | 1,62 | 1,62 |
| E | Glu | GAA | 1,5 | 1,5 | 1,5 | 1,5 | 1,49 | 1,5 | 1,5 | 1,49 | 1,5 | 1,5 | 1,42 | 1,5 | 1,5 | 1,49 |
| E | Glu | GAG | 0,5 | 0,5 | 0,5 | 0,51 | 0,51 | 0,51 | 0,5 | 0,51 | 0,5 | 0,5 | 0,58 | 0,51 | 0,5 | 0,51 |
| F | Phe | UUC | 0,75 | 0,75 | 0,72 | 0,74 | 0,75 | 0,74 | 0,75 | 0,76 | 0,75 | 0,75 | 0,74 | 0,74 | 0,73 | 0,74 |
| F | Phe | UUU | 1,25 | 1,25 | 1,28 | 1,26 | 1,25 | 1,26 | 1,25 | 1,24 | 1,25 | 1,25 | 1,26 | 1,26 | 1,27 | 1,26 |
| G | Gly | GGA | 1,64 | 1,6 | 1,57 | 1,62 | 1,6 | 1,63 | 1,64 | 1,59 | 1,6 | 1,6 | 1,59 | 1,62 | 1,58 | 1,62 |
| G | Gly | GGC | 0,37 | 0,37 | 0,37 | 0,39 | 0,36 | 0,39 | 0,37 | 0,4 | 0,37 | 0,37 | 0,36 | 0,39 | 0,37 | 0,39 |
| G | Gly | GGG | 0,69 | 0,71 | 0,74 | 0,7 | 0,72 | 0,7 | 0,69 | 0,71 | 0,71 | 0,71 | 0,74 | 0,7 | 0,72 | 0,71 |
| G | Gly | GGU | 1,3 | 1,31 | 1,33 | 1,28 | 1,32 | 1,28 | 1,31 | 1,3 | 1,31 | 1,31 | 1,3 | 1,29 | 1,33 | 1,28 |
| H | His | CAC | 0,46 | 0,48 | 0,47 | 0,46 | 0,47 | 0,45 | 0,46 | 0,46 | 0,48 | 0,48 | 0,5 | 0,46 | 0,49 | 0,46 |
| H | His | CAU | 1,54 | 1,52 | 1,53 | 1,54 | 1,53 | 1,55 | 1,54 | 1,54 | 1,52 | 1,52 | 1,5 | 1,54 | 1,51 | 1,54 |
| I | Ile | AUA | 0,94 | 0,95 | 0,95 | 0,93 | 0,96 | 0,93 | 0,94 | 0,93 | 0,94 | 0,95 | 0,95 | 0,93 | 0,94 | 0,93 |
| I | Ile | AUC | 0,61 | 0,59 | 0,59 | 0,62 | 0,6 | 0,63 | 0,61 | 0,64 | 0,6 | 0,59 | 0,6 | 0,62 | 0,6 | 0,62 |
| I | Ile | AUU | 1,45 | 1,46 | 1,47 | 1,45 | 1,44 | 1,44 | 1,45 | 1,43 | 1,45 | 1,46 | 1,45 | 1,45 | 1,46 | 1,45 |
| K | Lys | AAA | 1,44 | 1,43 | 1,44 | 1,45 | 1,43 | 1,44 | 1,44 | 1,44 | 1,43 | 1,43 | 1,43 | 1,45 | 1,43 | 1,45 |
| K | Lys | AAG | 0,56 | 0,57 | 0,56 | 0,55 | 0,57 | 0,57 | 0,56 | 0,56 | 0,57 | 0,57 | 0,57 | 0,55 | 0,57 | 0,55 |
| L | Leu | CUA | 0,82 | 0,84 | 0,83 | 0,82 | 0,84 | 0,81 | 0,83 | 0,83 | 0,83 | 0,84 | 0,84 | 0,82 | 0,85 | 0,81 |
| L | Leu | CUC | 0,43 | 0,43 | 0,44 | 0,43 | 0,43 | 0,44 | 0,43 | 0,42 | 0,43 | 0,43 | 0,42 | 0,44 | 0,44 | 0,43 |
| L | Leu | CUG | 0,39 | 0,39 | 0,38 | 0,39 | 0,4 | 0,39 | 0,39 | 0,41 | 0,39 | 0,4 | 0,38 | 0,39 | 0,39 | 0,4 |
| L | Leu | CUU | 1,25 | 1,23 | 1,23 | 1,26 | 1,23 | 1,26 | 1,24 | 1,24 | 1,23 | 1,22 | 1,21 | 1,25 | 1,22 | 1,25 |
| L | Leu | UUA | 1,87 | 1,89 | 1,87 | 1,87 | 1,86 | 1,85 | 1,87 | 1,88 | 1,89 | 1,89 | 1,97 | 1,86 | 1,88 | 1,87 |
| L | Leu | UUG | 1,24 | 1,22 | 1,25 | 1,23 | 1,25 | 1,25 | 1,24 | 1,24 | 1,22 | 1,22 | 1,18 | 1,24 | 1,22 | 1,24 |
| M | Met | AUG | 1 | 1 | 1 | 1 | 1 | 1 | 1 | 1 | 1 | 1 | 1 | 1 | 1 | 1 |
| N | Asn | AAC | 0,46 | 0,48 | 0,47 | 0,48 | 0,46 | 0,48 | 0,47 | 0,49 | 0,48 | 0,48 | 0,47 | 0,48 | 0,48 | 0,48 |
| N | Asn | AAU | 1,54 | 1,52 | 1,53 | 1,52 | 1,54 | 1,52 | 1,54 | 1,51 | 1,52 | 1,52 | 1,53 | 1,52 | 1,52 | 1,52 |
| P | Pro | CCA | 1,17 | 1,14 | 1,12 | 1,16 | 1,11 | 1,13 | 1,17 | 1,12 | 1,14 | 1,13 | 1,08 | 1,16 | 1,11 | 1,15 |
| P | Pro | CCC | 0,89 | 0,96 | 1,03 | 0,94 | 0,98 | 0,96 | 0,88 | 0,92 | 0,96 | 0,97 | 0,96 | 0,93 | 0,96 | 0,93 |
| P | Pro | CCG | 0,47 | 0,48 | 0,43 | 0,44 | 0,46 | 0,46 | 0,47 | 0,49 | 0,48 | 0,48 | 0,54 | 0,44 | 0,49 | 0,44 |
| P | Pro | CCU | 1,47 | 1,42 | 1,42 | 1,46 | 1,45 | 1,45 | 1,49 | 1,47 | 1,42 | 1,41 | 1,42 | 1,47 | 1,44 | 1,48 |
| Q | Gln | CAA | 1,52 | 1,53 | 1,54 | 1,53 | 1,5 | 1,54 | 1,52 | 1,52 | 1,52 | 1,52 | 1,53 | 1,53 | 1,51 | 1,53 |
| Q | Gln | CAG | 0,48 | 0,47 | 0,46 | 0,47 | 0,5 | 0,46 | 0,48 | 0,48 | 0,48 | 0,48 | 0,47 | 0,47 | 0,49 | 0,47 |
| R | Arg | AGA | 1,87 | 1,85 | 1,87 | 1,86 | 1,83 | 1,87 | 1,86 | 1,84 | 1,85 | 1,85 | 1,86 | 1,87 | 1,86 | 1,87 |
| R | Arg | AGG | 0,67 | 0,68 | 0,73 | 0,67 | 0,73 | 0,67 | 0,67 | 0,69 | 0,68 | 0,68 | 0,71 | 0,67 | 0,7 | 0,68 |
| R | Arg | CGA | 1,33 | 1,31 | 1,3 | 1,3 | 1,26 | 1,31 | 1,33 | 1,26 | 1,31 | 1,31 | 1,27 | 1,3 | 1,3 | 1,3 |
| R | Arg | CGC | 0,33 | 0,37 | 0,37 | 0,36 | 0,38 | 0,36 | 0,33 | 0,41 | 0,36 | 0,37 | 0,37 | 0,36 | 0,39 | 0,36 |
| R | Arg | CGG | 0,46 | 0,49 | 0,47 | 0,47 | 0,49 | 0,45 | 0,45 | 0,5 | 0,49 | 0,48 | 0,49 | 0,46 | 0,49 | 0,47 |
| R | Arg | CGU | 1,35 | 1,31 | 1,27 | 1,35 | 1,3 | 1,34 | 1,37 | 1,3 | 1,31 | 1,31 | 1,3 | 1,35 | 1,26 | 1,33 |
| S | Ser | AGC | 0,33 | 0,31 | 0,34 | 0,32 | 0,36 | 0,32 | 0,32 | 0,33 | 0,32 | 0,32 | 0,32 | 0,31 | 0,32 | 0,32 |
| S | Ser | AGU | 1,19 | 1,19 | 1,18 | 1,2 | 1,18 | 1,19 | 1,19 | 1,17 | 1,2 | 1,19 | 1,18 | 1,19 | 1,2 | 1,2 |
| S | Ser | UCA | 1,27 | 1,26 | 1,28 | 1,23 | 1,34 | 1,25 | 1,26 | 1,24 | 1,26 | 1,26 | 1,21 | 1,23 | 1,26 | 1,22 |
| S | Ser | UCC | 1,01 | 1,04 | 1,07 | 1,02 | 1,02 | 1,03 | 1 | 1,04 | 1,04 | 1,04 | 1,04 | 1,02 | 1,03 | 1,02 |
| S | Ser | UCG | 0,55 | 0,56 | 0,5 | 0,6 | 0,47 | 0,59 | 0,57 | 0,58 | 0,56 | 0,56 | 0,63 | 0,61 | 0,56 | 0,61 |
| S | Ser | UCU | 1,65 | 1,63 | 1,64 | 1,63 | 1,63 | 1,63 | 1,66 | 1,64 | 1,63 | 1,63 | 1,61 | 1,64 | 1,63 | 1,62 |
| T | Thr | ACA | 1,21 | 1,24 | 1,27 | 1,22 | 1,22 | 1,22 | 1,22 | 1,19 | 1,24 | 1,24 | 1,21 | 1,22 | 1,24 | 1,22 |
| T | Thr | ACC | 0,73 | 0,72 | 0,74 | 0,74 | 0,72 | 0,75 | 0,72 | 0,74 | 0,72 | 0,72 | 0,75 | 0,74 | 0,72 | 0,74 |
| T | Thr | ACG | 0,5 | 0,5 | 0,44 | 0,51 | 0,53 | 0,51 | 0,5 | 0,54 | 0,5 | 0,5 | 0,52 | 0,51 | 0,48 | 0,51 |
| T | Thr | ACU | 1,56 | 1,54 | 1,54 | 1,53 | 1,53 | 1,53 | 1,56 | 1,53 | 1,54 | 1,54 | 1,53 | 1,53 | 1,56 | 1,54 |
| V | Val | GUA | 1,5 | 1,51 | 1,49 | 1,5 | 1,49 | 1,51 | 1,5 | 1,48 | 1,51 | 1,5 | 1,51 | 1,49 | 1,51 | 1,5 |
| V | Val | GUC | 0,48 | 0,49 | 0,44 | 0,48 | 0,46 | 0,48 | 0,48 | 0,49 | 0,49 | 0,49 | 0,49 | 0,48 | 0,49 | 0,48 |
| V | Val | GUG | 0,58 | 0,57 | 0,6 | 0,57 | 0,59 | 0,57 | 0,58 | 0,59 | 0,57 | 0,57 | 0,57 | 0,58 | 0,57 | 0,58 |
| V | Val | GUU | 1,44 | 1,43 | 1,47 | 1,45 | 1,46 | 1,44 | 1,45 | 1,45 | 1,43 | 1,44 | 1,42 | 1,45 | 1,44 | 1,45 |
| W | Trp | UGG | 1 | 1 | 1 | 1 | 1 | 1 | 1 | 1 | 1 | 1 | 1 | 1 | 1 | 1 |
| Y | Tyr | UAC | 0,41 | 0,4 | 0,4 | 0,42 | 0,41 | 0,42 | 0,41 | 0,4 | 0,4 | 0,4 | 0,42 | 0,42 | 0,41 | 0,42 |
| Y | Tyr | UAU | 1,59 | 1,6 | 1,6 | 1,58 | 1,59 | 1,58 | 1,59 | 1,6 | 1,6 | 1,6 | 1,58 | 1,58 | 1,59 | 1,58 |
