# Supplementary material for: Phylogenomics, divergence time estimation, and biogeography of Iris species from Kazakhstan using plastome sequence analysis
Source: Front Plant Sci. 2026 Jun 17;17:1860819. doi: 10.3389/fpls.2026.1860819 (PMC13318877; doi:10.3389/fpls.2026.1860819)
Supplement: Supplementary file 7 [file Table7.docx]

**Supplementary Table S7.** Comparison of biogeographic models

| alt | null | LnL  alt | LnL  null | DF  alt | DF  null | DF | Dstatistic | pval | test | tail | AIC1 | AIC2 | AICwt1 | AICwt2 | AIC weight ratio model1 | AIC weight ratio model2 |
| --- | --- | --- | --- | --- | --- | --- | --- | --- | --- | --- | --- | --- | --- | --- | --- | --- |
| DEC+J | DEC | -199.5 | -201.1 | 3 | 2 | 1 | 3.14 | 0.077 | chi-squared | one-tailed | 405 | 406.1 | 0.64 | 0.36 | 1.76 | 0.57 |
| DIVALIKE+J | DIVALIKE | -214.9 | -215 | 3 | 2 | 1 | 0.32 | 0.57 | chi-squared | one-tailed | 435.8 | 434.1 | 0.30 | 0.70 | 0.43 | 2.32 |
| BAYAREALIKE+J | BAYAREALIKE | -182 | -196.7 | 3 | 2 | 1 | 29.42 | 5.8e-08 | chi-squared | one-tailed | 369.9 | 397.4 | 1.00 | 1.1e-06 | 901607 | 1.1e-06 |
