# Supplementary material for: Phylogenomics, divergence time estimation, and biogeography of Iris species from Kazakhstan using plastome sequence analysis
Source: Front Plant Sci. 2026 Jun 17;17:1860819. doi: 10.3389/fpls.2026.1860819 (PMC13318877; doi:10.3389/fpls.2026.1860819)
Supplement: Supplementary file 8 [file Table8.docx]

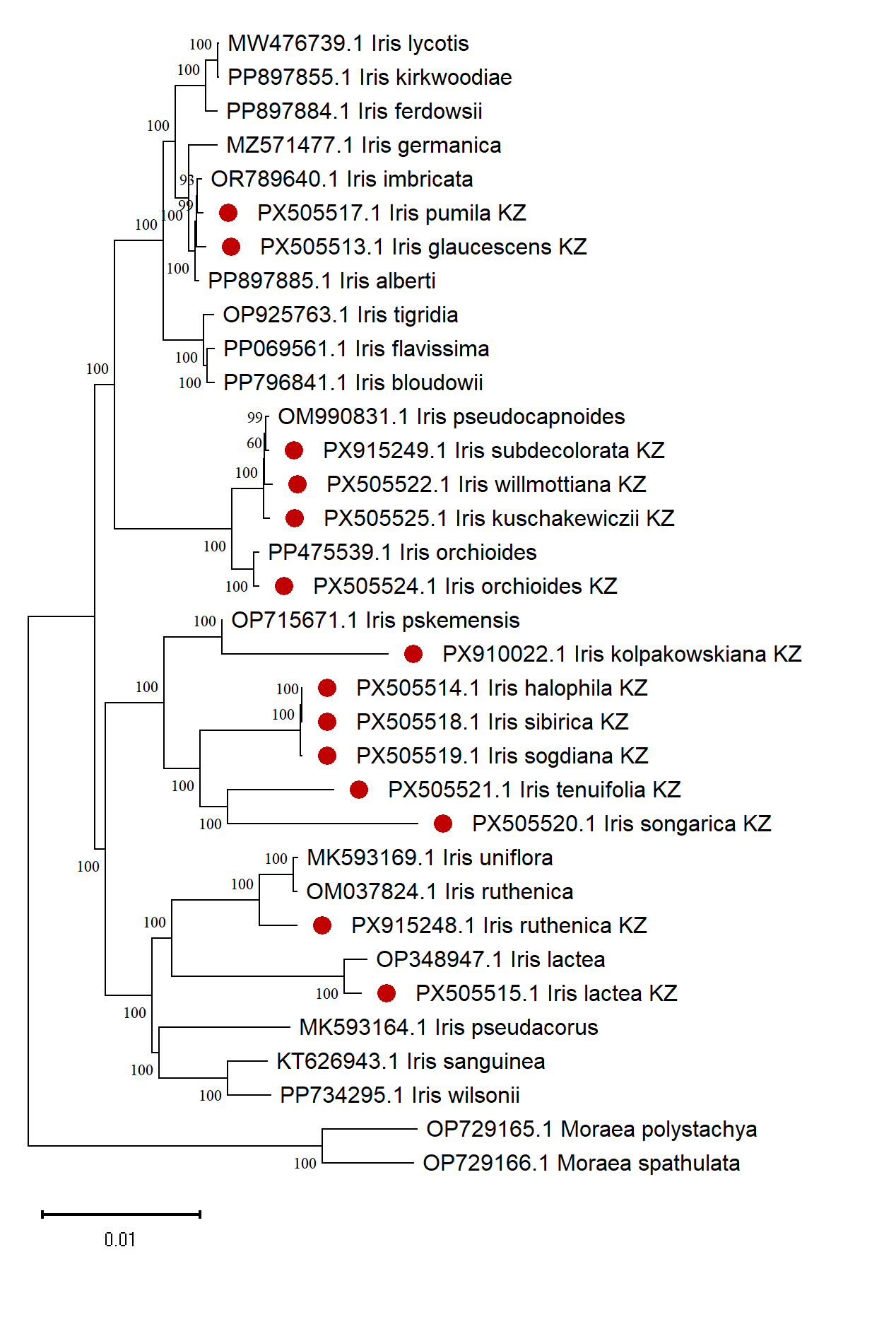


**Figure S1.** Phylogenetic tree of Iris species constructed using the Neighbor-Joining (NJ) method based on complete plastome nucleotide sequences. Numbers at the nodes indicate bootstrap values. The species sequenced in this study are highlighted with red circles.
